# Development of Bioinspired Functional Chitosan/Cellulose Nanofiber 3D Hydrogel Constructs by 3D Printing for Application in the Engineering of Mechanically Demanding Tissues

**DOI:** 10.3390/polym13101663

**Published:** 2021-05-20

**Authors:** Arnaud Kamdem Tamo, Ingo Doench, Lukas Walter, Alexandra Montembault, Guillaume Sudre, Laurent David, Aliuska Morales-Helguera, Mischa Selig, Bernd Rolauffs, Anke Bernstein, Daniel Hoenders, Andreas Walther, Anayancy Osorio-Madrazo

**Affiliations:** 1 Laboratory for Sensors, Institute of Microsystems Engineering IMTEK, University of Freiburg, 79110 Freiburg, Germany; arnaud.kamdem@imtek.uni-freiburg.de (A.K.T.); ingo.doench@imtek.uni-freiburg.de (I.D.); lukas.walter@imtek.uni-freiburg.de (L.W.); 2 Freiburg Materials Research Center—FMF, University of Freiburg, 79104 Freiburg, Germany; 3 Freiburg Center for Interactive Materials and Bioinspired Technologies—FIT, University of Freiburg, 79110 Freiburg, Germany; 4 Ingénierie des Matériaux Polymères IMP UMR 5223—CNRS, Université Claude Bernard Lyon 1, Université de Lyon, CEDEX, 69622 Villeurbanne, France; alexandra.clayer-montembault@univ-lyon1.fr (A.M.); guillaume.sudre@univ-lyon1.fr (G.S.); laurent.david@univ-lyon1.fr (L.D.); 5 Chemical Bioactive Center CBQ, Molecular Simulation and Drug Design Group, Central University of Las Villas, Santa Clara 50400, Cuba; aliuska@uclv.edu.cu; 6 Center for Tissue Replacement, Regeneration & Neogenesis—G.E.R.N., Department of Orthopedics and Trauma Surgery, University of Freiburg, 79108 Freiburg, Germany; mischa.selig91@gmail.com (M.S.); berndrolauffs@googlemail.com (B.R.); anke.bernstein@uniklinik-freiburg.de (A.B.); 7 Department of Chemistry, University Mainz, 55128 Mainz, Germany; daniel.hoenders@uni-mainz.de (D.H.); andreas.walther@uni-mainz.de (A.W.)

**Keywords:** hydrogel 3D printing, polymer composites, chitosan, cellulose nanofibers, X-ray synchrotron scattering, micromechanics, tissue engineering

## Abstract

Soft tissues are commonly fiber-reinforced hydrogel composite structures, distinguishable from hard tissues by their low mineral and high water content. In this work, we proposed the development of 3D printed hydrogel constructs of the biopolymers chitosan (CHI) and cellulose nanofibers (CNFs), both without any chemical modification, which processing did not incorporate any chemical crosslinking. The unique mechanical properties of native cellulose nanofibers offer new strategies for the design of environmentally friendly high mechanical performance composites. In the here proposed 3D printed bioinspired CNF-filled CHI hydrogel biomaterials, the chitosan serves as a biocompatible matrix promoting cell growth with balanced hydrophilic properties, while the CNFs provide mechanical reinforcement to the CHI-based hydrogel. By means of extrusion-based printing (EBB), the design and development of 3D functional hydrogel scaffolds was achieved by using low concentrations of chitosan (2.0–3.0% (*w/v*)) and cellulose nanofibers (0.2–0.4% (*w/v*)). CHI/CNF printed hydrogels with good mechanical performance (Young’s modulus 3.0 MPa, stress at break 1.5 MPa, and strain at break 75%), anisotropic microstructure and suitable biological response, were achieved. The CHI/CNF composition and processing parameters were optimized in terms of 3D printability, resolution, and quality of the constructs (microstructure and mechanical properties), resulting in good cell viability. This work allows expanding the library of the so far used biopolymer compositions for 3D printing of mechanically performant hydrogel constructs, purely based in the natural polymers chitosan and cellulose, offering new perspectives in the engineering of mechanically demanding hydrogel tissues like intervertebral disc (IVD), cartilage, meniscus, among others.

## 1. Introduction

There is an increasing interest in the development of 3D complex functional architectures with appropriate biomaterials and cells, in particular with the strategy of mimicking the cellular microenvironment of native tissues. Three-dimensional printing has emerged as a powerful tool for tissue engineering, which applies additive manufacturing to biofabricate 3D tissue-resembling objects with a high degree of spatial organization [1,2]. A layer-by-layer deposition of materials [1], called (bio)inks, is performed in specifically designed 3D shapes [3,4,5]. It can potentially impact patient care through fabrication of tissue substitutes for implantation and regeneration, or for drug and toxicity screening [6]. Engineering of polymer biomaterials with controlled microstructure approaching tissue functionalities, while serving as model to investigate cell behavior in more relevant 3D mimicking environments, is challenging [7]. The biomaterial and the cell types are key components, which can also impact the biofabrication process [8,9]. The development of bioinks, with good printability and bioactive properties, controlling cellular fate, still needs to be improved in order to advance the translation into the clinic. In a first step, to provide the proper physical, chemical, and biological cues to the cells, bioprinting of biocompatible biomaterials needs to be finely studied, since they directly act on cellular adhesion, differentiation, and proliferation. Three-dimensional scaffolds with interconnected pore network microstructures have been widely studied and shown to ensure cell viability and colonization and the diffusion of nutrients [10,11]. Before scaffold formation, (bio)ink characteristics should include appropriate viscosity, printability, biocompatibility, enhanced cell adhesion, mechanical performance, and, finally, biodegradability, if desired. A relatively limited number of biopolymer hydrogels are available as bioinks. Among them, the alginate-based systems are the most investigated ones [12], which have been tested for viability of fibroblasts, stem cells, chondrocytes, osteogenic activity support, neural tissue construction, etc. [13]. Nevertheless, alginate does not bind strongly with cells, often leading to improper cellular interactions. In vivo, it may induce strong inflammatory response and is relatively fast biodegraded. Gelatin is also commonly used due to its mild physical gelation triggered by temperature change [14]. Hydrogels of collagen, gelatin modified with methacrylate moieties, agarose, chitosan, carboxymethylchitosan, polycaprolactone (PCL), silk fibroin, hyaluronic acid (HA) [15,16], have been 3D printed, even with cellulose nanofibers [17], but not all of them were fabricated for tissue engineering applications.

There are three main methodologies used for 3D bioprinting: inkjet, extrusion-based, and light-assisted bioprinting. Microextrusion (EBB) can be performed with a broad viscosity range of dispensable materials by using inexpensive setups [18,19]. Depending on ink and plotting device, thin lines of ink with a width range from around 45 to 1200 μm can be printed by EBB. Liquids with low to very high viscosity (until around 10^7^ mPa.s) are reported to be printable via extrusion. Specially, the polymer ink needs to be in “liquid” phase prior to extrusion to avoid nozzle clogging, and must gel very fast after extrusion, to become a stable 3D free-standing object. Thus, not all biomaterials are printable by EBB. In this work, EBB is the method of choice to fabricate mechanically performant 3D fiber-reinforced hydrogel constructs only constituted of natural compounds like chitosan and cellulose, without biopolymer modification or addition of any chemical crosslinker, for a vast range of applications in tissue engineering.

In view of the exceptional properties of chitosan hydrogels for tissue engineering [20,21,22,23,24,25], we propose here the 3D printing of bioinspired functional chitosan (CHI) hydrogels filled with non-modified cellulose nanofibers (CNF). Soft tissues like the intervertebral dics, skin, among others, are commonly complex fiber-reinforced hydrogel composites [21,22,26,27]. Chitosan is a family of chitin-derived cationic polysaccharides, consisting of β-(1-4)-glucosamine and *N*-acetyl-glucosamine units [28,29,30,31,32,33]. It exhibits excellent biological properties like biocompatibility, biodegradability, bacteriostaticity, and fungistaticity, and promotes wound healing, cell proliferation, tissue repair [21,22,25,34,35,36,37,38]. Cellulose is the main component of plant biomass and most abundant biopolymer worldwide. Its chains naturally form a highly crystalline structure (namely cellulose I allomorph), assembled into microfibrils [39,40,41,42,43]. The unique crystalline properties of the processed native cellulose nanofibrils, combined with their high aspect ratio and renewability, have placed the cellulose nanofibers (CNF) as a green nanoreinforcement alternative for the design of environmentally friendly and high mechanical performance composites [39,44,45,46]. Specially, the nano/microfibrillated cellulose (MFC) can be produced from the peeling of the native fibers into a network of hairy fibrils [47,48,49]. As a nanomaterial, the question on CNF toxicity and environmental impact has been addressed [22]. Low toxicity has been reported, according to ecotoxicological, cytotoxicity and proinflammatory response studies [50,51,52]. Cellulose nanofibers have also been proposed for drug delivery systems [53], reinforcement for biomaterials [21,22,54,55,56], protein immobilization [57], etc. In hydrogel biomaterials, the use of CNFs is promising-in addition to their mechanical performance, CNFs form a network with high water retention, they are biocompatible and can yield transparent biomaterials [21,22,54,58]. Besides, cellulose nanofibers can be oriented within hydrogels. Osorio et al. [39] performed pioneer works where cellulose whisker nanocrystals were oriented in bulk polysaccharide hydrogel matrices by uniaxial stretching under controlled humidity, to yield anisotropic hydrogels.

The goal of this work is the development by microextrusion of bioinspired mechanically performant fiber-reinforced 3D hydrogel scaffolds, of completely natural polymers like chitosan, without any chemical crosslinking to achieve its gelation, and using as nanoreinforcement non-modified cellulose nanofibers, which hydrogel composites should find application in the engineering of mechanically demanding hydrogel tissues like intervertebral disc (IVD) [21,22,59,60], cartilage, meniscus, etc. Different natural polymers have been explored in hydrogel biomaterials to design provisional supports for regeneration/repair of intervertebral disc and cartilaginous tissues; among them, collagen [61], hyaluronic acid (HA) and its derivatives [61,62,63,64], chondroitin sulfate [64], agarose [65], alginate [66], chitosan and its derivatives [21,22,25], cellulose and its derivatives [21,22,67]. Doench et al. [21,22] prepared bulk chitosan hydrogels reinforced with CNFs for the regeneration and repair of both the *annulus fibrosus* (AF)and the *nucleus purposus* (NP) regions of the intervertebral disc. They concluded that the addition of CNF significantly improves the mechanical properties of the composite hydrogels. Moreover, the in situ gelation of the CHI/CNF precursor viscous suspension could be used as non-invasive injectable applications in disc nucleosupplementation [22]. Additionally, they carried out ex vivo experiments in porcine models, also evidencing that the implantation of CHI/CNF composite hydrogels within fenestrated (defective) discs helps to restore their biomechanics [21,22]. Specially, the performance for *annulus fibrosus* (AF) tissue repair of distinct hydrogels based on HA, chondroitin sulfate, collagen has been evaluated in vivo applying rabbit, porcine, or bovine models [61,62,63,64]. Although hydrogels implantation has been related to an upregulation of extracellular matrix (ECM) genes such as Col1, Col2, and Decorin and catabolic matrix metalloproteases MMP13 and MMP3, nucleotomy, and implant administration resulted in localized annular damage with AF inflammation and scarring, impairing a proper tissue regeneration [61]. Therefore, other strategies focused on keeping disc integrity, preventing AF damage and NP reherniation. In this context, treatment of the *nucleus pulposus* with a liquid HA-based implant was proposed, which aimed to fill irregularly shaped defects through in situ polymerization yielding a strain resistant hydrogel [63]. A comparable approach investigated a minimal invasive shape-memory *annulus fibrosus* closing device of poly(d,l-lactide–*co*–trimethylene carbonate) (PLA-TMC) that revealed an elastic modulus of 1.7–2.5 MPa, ranging in the modulus of human AF tissue [68]. In conclusion, there is increasing interest in designing a non-inflammatory, mechanically performant, and well-integrated hydrogel for intervertebral disc repair, possibly with AF tissue sealing capacity, to ensure long-term regeneration of the IVD while preventing further degeneration.

In this work, the interest of cellulose is to ensure the mechanical performance needed in the pre-gelation state to deposit free-standing hydrogel constructs, while achieving fiber-filled bioinspired hydrogel biomaterials. The development strategy was based on three main factors to obtain printable formulations: viscosity adjustment; ink flow prior to extrusion, and printed material-specific gelation and mechanical performance. In terms of nomenclature, it is worth noticing that the name “bioink” sometimes is used, as referred to the achievement of 3D printed hydrogel constructs for cell seeding and growing, even if biological bodies themselves were not directly incorporated in the initial ink. Different CHI/CNF ink compositions will be considered in this work. To evaluate the suitability of the 3D printed CHI/CNF hydrogel constructs for tissue engineering, cell growth and migration will be evaluated in 3D cell culture studies. The CHI/CNF composition and processing parameters will be optimized in terms of 3D printability and good quality of constructs (microstructure and mechanics), resulting in good cell viability.

## 2. Materials and Methods

### 2.1. Chitosan Source and Characterization

Chitosan from squid pen chitin was supplied by Mahtani Chitosan (Veraval, India; Batch type 144). The chitosan degree of acetylation (DA) was determined by ^1^H NMR spectroscopy following the methodology of Hirai et al. [69,70]. The measurement was performed on a Bruker ALS 300 spectrometer (Bruker GmbH, Ettlingen, Germany) (300 MHz for ^1^H) at 298 K, which revealed a low DA of 2.5%. The CHI molecular weight was determined by size exclusion chromatography (SEC) coupled to multiangle laser light scattering (MALLS) at the Chromatography Center of Institute of Chemistry of Lyon (ICL) [70,71,72,73]. To this end, chitosan solutions at 0.1% (*w/v*) were prepared in an acetic acid/ammonium acetate buffer pH = 4.5 (AcOH (0.2 M)/AcONH4 (0.15 M)), which was used as an eluent. Then, they were filtered through 0.45 μm pore size membranes (Millipore, Merck KGaA, Darmstadt, Germany). The chromatographic equipment was composed of an IsoChrom LC pump (Spectra-Physics, Santa Clara, CA, USA) connected to a Protein Pack 200 SW (Waters GmbH, Eschborn, Germany) column and a TSK gel G6000 PWXL. A multiangle laser light scattering detector DAWNDSP (Wyatt Technology Europe GmbH, Dernbach, Germany), operating at 632.8 nm, was coupled online to a WATERS 410 differential refractometer. The chitosan number (Mn) and weight-average molecular weights (Mw) determined by SEC/MALLS were 4.10 × 10^5^ g/mol (± 6.4%) and 6.11 × 10^5^ g/mol (±9.6%), respectively, which yield a polydispersity index *I*_p_ = *M*_w_/*M*_n_ = 1.49 (±11.6%).

### 2.2. Cellulose Nanofibers

The used cellulose nanofiber type was nanofibrillated cellulose. Cellulose nanofiber (CNF) suspensions were obtained from bleached pine sulfite dissolving pulp at the Centre Technique du Papier (CTP, Grenoble, France), by a mechanoenzymatic method adapted from Pääkkö et al. [74]. Before 1 h incubation at 50 °C with a solution of endoglucanase FiberCare R^®^ (Novozyme, Bagsvaerd, Denmark) at pH 5.0, the pulp was refined at 4.5% consistency with a 12″ single disk refiner for 25 min. The digested samples were further refined to obtain a pulp suspension of Schopper-Riegler (SR) number higher than 80 SR and mean fiber length lower than 300 µm. Fiber suspensions at 2% (*w/w*) were collected with an Ariete homogenizer (Montigny le Bretonneux, France), involving one pass at 1000 bar, followed by 3 passes at 1500 bars. The obtained CNFs displayed a surface charge density of 40–80 mmol/kg and were weakly charged with carboxylate moieties.

### 2.3. Transmission Electron Microscopy

Morphology and dimensions of the cellulose nanofibers were investigated by transmission electron microscopy (TEM). Drops of a 0.001% (*w/v*) CNF suspension were deposited on carbon coated copper grids (CF400-CU Carbon Film, 400 Mesh Copper grids), which were previously cleaned with plasma to get rid of surface contamination and make the grid more adhesive towards the sample. The samples were negatively stained with 2% (*w/v*) uranyl acetate in suspension. Then, samples were allowed to dry and were observed in the TEM microscope, model Zeiss LEO 912 Omega (Carl Zeiss Microscopy GmbH, Jena, Germany), operating at 80 kV.

### 2.4. Preparation of Chitosan/Cellulose Nanofiber Viscous Inks

Chitosan (CHI) viscous solutions: A stoichiometric amount of acetic acid was added to chitosan powder dispersed in MilliQ water, as to protonate the free amine groups present in the glucosamine units of chitosan (accounting for a chitosan degree of acetylation (DA) value of 2.5%) and thereby allow the CHI complete solubilization. The mixture was left under mechanical stirring overnight. Chitosan aqueous solutions at 2%, 3%, and 4% (*w/v*) were prepared, named as CHI2, CHI3, and CHI4 formulations, respectively.

Cellulose nanofiber-filled CHI viscous suspensions: The CHI powder was firstly mixed with CNFs aqueous suspension in MilliQ water and the dispersions were sonicated with a SONOPULS Ultrasonic homogenizer (Bandelin electronic GmbH, Berlin, Germany) for 5 min at 40% amplitude. Then, a stoichiometric amount of acetic acid was added to completely solubilize the CHI contained in the mixture. The obtained suspension was mechanically stirred overnight to ensure a good dispersion of CNFs within the CHI solution. Viscous ink formulations, with CHI concentration of 2%, 3%, or 4% (*w/v*) and cellulose nanofibers (CNF) contents of 0.4%, 0.5%, or 0.6% (*w/v*) were obtained.

#### Rheological Behavior

A rotational rheometer AR-2000 (TA Instruments, New Castle, DE, USA) fitted with a cone and a plate flow geometry was used to characterize the rheological behavior of the CHI/CNF viscous inks at 25 °C, with a gap size of 116 µm and a solvent trap to prevent its drying or evaporation. The cone-plate geometry (25 mm diameter; 4°) allows one to ensure a uniform shearing to the sample. The analysis was performed in triplicate in the continuous mode in a shear rate range from 0.005 to 1000 s^−1^ and the equilibrium time was set at 15 s. TA Instruments TRIOS software was used in the measurements. Finally, the flow diagrams of the CHI/CNF ink formulations were obtained, namely the plots of the steady-state shear viscosity vs. shear rate.

### 2.5. 3D Printing of Cellulose Nanofiber-Filled Chitosan Hydrogel Scaffolds

The 3D printing of scaffolds of chitosan/cellulose nanofibers hydrogels was performed with the 3D-Discovery Evolution bioprinter device (RegenHU, Villaz-St-Pierre, Switzerland), consisting of an x–y–z-axis positioning system with a tool charger equipped with several printhead stations, and a building platform. The 3D structures were printed using a CAD software controlled xyz motion-system that guides the tip position. Multilayer square-shaped porous CHI/CNF hydrogel structures were 3D printed by microextrusion using a pressure controlled direct-ink-writing system.

After preparing the inks as above (i.e., CHI solutions and CHI/CNF suspensions) they were centrifuged for 20 min at 2000 rpm, to eliminate air bubbles before microextrusion. In the 3D-Discovery bioprinter, the inks were loaded in a syringe (Nordson EFD, Feldkirchen, Germany) mounted in a dispensing adaptor and extruded through precision conic dispense tips with inner diameter of 250 or 410 μm (Nordson EFD, Feldkirchen, Germany), with a printing speed of 40 mm·s^−1^ under applying a given pressure (0.15–0.77 bar). Scaffolds were shaped as nets of 30 × 30 mm^2^ in size, composed by the alternation of 4 orthogonal layers, each constituted of parallel hydrogel filaments printed with 0.85 mm interspace. Hydreogel scaffolds were directly printed in Petri dishes containing aqueous 2 M NaOH for chitosan neutralization, which was used as coagulation bath. The distance between the extrusion needle tip and the Petri dish surface was set at 3 mm, by using a calibration laser integrated to the 3D-Discovery Evolution bioprinter device. After a neutralization time of 15 min, scaffolds were taken out from the alkaline bath and washed several times with MilliQ water until neutrality. Chitosan/cellulose nanofiber hydrogel filaments and 3D scaffolds were printed using the above protocol with ink formulations containing CHI at a concentration of 2 or 3% (*w/v*), and a CNF content of 0.4% (*w/v*). Table 1 summarizes the varied CHI/CNF ink compositions with the different inner diameters (ID) of the extrusion needles and extrusion pressure used in the direct-ink-writing process in the bioprinter device.

#### 2.5.1. Scanning Electron Microscopy

The freeze-dried CHI/CNF printed hydrogel scaffolds were carefully fractured and gold sputtered in a Polaron SC 7640 (VG Microtech, East Sussex, UK), and observed by scanning electron microscopy (SEM) (Amray Inc., Bedford, MA, USA) at an accelerating voltage of 15 kV. It is worth noticing that with this technique the analysis was performed in the freeze-dry state. Nevertheless, it allowed to get insight into the dispersion of the CNFs in the dry scaffold composites, which were originated from the hydrogel processing.

#### 2.5.2. Microtensile Testing

The hydrogels of all CHI/CNF ink formulations shown in Table 1 were printed in a single horizontal layer with an interspace of 2 mm, to characterize the hydrogel filament resolution and mechanical properties. These latter were investigated using a microtensile testing device. Hydrogel filaments were cautiously cut by using a razorblade to obtain a length of approximately 8 mm. The cut filaments were glued onto a foliar frame with a test span of about 7 mm by using cyanoacrylate glue (Loctite^®^ 454, Henkel AG & Co, Munich, Germany). Tests were conducted under controlled relative humidity (RH) of 45%, at a constant crosshead displacement speed of 8 µm/s, and the applied force (*F*) was measured by a load cell with a maximum capacity of 50 N. The nominal stress σ was calculated as the ratio of the applied force F to the initial cross sectional area *A* of the hydrogel filament (σ = *F*/*A*), and the nominal strain ε was expressed as the ratio of the extension of the hydrogel filament respect to its initial length l_0_ (ε = Dl/l_o_ = (l − l_0_)/l_0_). The Young’s modulus (E), ultimate stress (σ_b_), and strain at break (ε_b_) were determined from the obtained stress–strain curves, considering at least six measurement replicates (*n* = 6) for each printed formulation.

#### 2.5.3. Wide and Small-Angle X-ray Synchrotron Scattering (WAXS and SAXS). In Situ Microtensile Testing

X-ray synchrotron scattering analyses were performed at the microfocus beamline mySpot, BESSY II at the Helmholtz-Zentrum Berlin HZB (Germany) and at the beamline BM02/D2AM at the European Synchrotron Radiation Facility ESRF (Grenoble, France). At Bessy II, data were collected at a wavelength λ = 1.0 Å, which setup allowed simultaneously measuring small (SAXS) and wide-angle X-ray scattering (WAXS) using a two-dimensional MARCCD detector. At this microfocus beamline, the synchrotron X-ray beam had a diameter of around 10 μm, which passed through the CHI/CNF printed hydrogel filament placed in a holder with Kapton foil windows, allowing for in situ tensile testing of the hydrogel biomaterial while recording SAXS and WAXS signals. At the ESRF, SAXS data were collected at a wavelength λ = 0.78 Å using a CCD detector (Roper Scientific GmbH, Ottobrunn, Germany). Both at Bessy II and at the ESRF, the silver behenate was used as standard to calibrate the scattering vector *q*-range, and transmission corrections and background subtraction were performed in the SAXS/WAXS data treatment.

### 2.6. 3D Cell Culture of Fibroblasts in the Printed Hydrogel Scaffolds

To evaluate the suitability of the CNF/CHI 3D printed hydrogels for tissue engineering, fibroblast cells were cultured in the 3D hydrogel scaffolds, as 3D printed and washed, without any hydrogel drying step before cell cultivation. Cultures of 3T3 cells developed using the NIH Swiss mouse embryo fibroblasts were performed (murine fibroblast, strain: NIH/Swiss). Cells were grown in T75 (75 cm^2^) cell culture flasks (Sarstedt, Nümbrecht, Germany). The cells were cultured in Dulbecco’s modified Eagle medium (DMEM) supplemented with 2 mM l-glutamine and 10% fetal bovine serum (FBS) (Gibco, Thermo Fisher Scientific, Leicestershire, UK) at 37 °C in a humidified atmosphere of 5% CO_2_ for 1 week. Upon 90% confluence, cells were rinsed twice with phosphate-buffered saline (PBS) (Gibco, Thermo Fisher Scientific, Leicestershire, UK) followed by detachment with trypsin/ethylene diamine tetra acetic acid (EDTA) for 5 min and neutralization with the corresponding cell culture medium. After detachment, cells were spun down in a centrifuge for 5 min at 110 rcf (Rotor F-45-30-11, Eppendorf 5417R, Hamburg, Germany). The supernatant was discarded and cells were diluted into the culture medium. The 3D printed CHI/CNF hydrogel scaffolds were put in 6-well cell culture plates, covering the whole well surface for subsequent use for cell growth. To this end, 500 μL of suspension of the cells in culture medium as above were added on the 3D printed CHI/CNF hydrogel scaffolds, considering a starting loading average of 10^5^ cells per well. The NIH/3T3 fibroblasts seeded in triplicate in scaffolds of different CHI concentrations and CNF contents were kept at 37 °C in CO_2_ incubation. Cells seeded in empty wells (i.e., without 3D-printed hydrogels) were used as a control.

#### Live/Dead Cell Viability Assay

The viability of the cells was qualitatively evaluated to get an indication of whether fibroblasts survive and grow in the CHI/CNF hydrogel scaffolds. Cultivation of at least 6 days was possible and proliferation was observed. The cells were inspected for viability by fluorescent staining with a live/dead staining kit: calcein AM/ethidium-homodimer-1, LIVE/DEAD™ viability/cytotoxicity kit, (Thermo Fisher Scientific, Leicestershire, UK). After 1, 3, and 6 days of cell culture in the 3D hydrogel scaffolds as described above, a cell washing step was performed with Hank’s balanced salt solution (HBSS, Gibco, Thermo Fisher Scientific, Leicestershire, UK) and the freshly prepared LIVE/DEAD solution was added to each sample. Samples were kept at incubation conditions for 15 min. After staining, the wells were imaged using a confocal laser-scanning microscope (Leica TCS SPE, Wetzlar, Germany). As the LIVE indicator, calcein AM marks cell cytoplasm in green fluorescence; and as the DEAD indicator, ethidium homodimer-1 stains cell nucleus in red fluorescence.

Cell counting: Image analysis of the LIVE/DEAD cell viability assay micrographs was performed with Fiji (v1.52u), an ImageJ-based program [75]. Images were converted to 8-bit and the contrast was normalized via ‘histogram normalization’ command. The trainable weka segmentation tool was then used for cell segmentation [76]. Cells were counted using the Fiji “find maxima” command with a constant noise level to distinguish cell maxima vs. background noise. The results were exported to Excel for further statistical analyses.

Statistical analysis: All cell counting data were expressed as mean ± standard deviation (SD). Statistical analysis was performed by the one-way analysis of variance (ANOVA) using the software STATISTICA 10.0 (StatSoft Inc: Tulsa, OK, USA, 2011), followed by the Tukey’s HSD post hoc test if significant differences were found (*p* < 0.05) in the ANOVA.

## 3. Results and Discussion

### 3.1. Cellulose Nanofibers Microstructure

**Figure 1:**
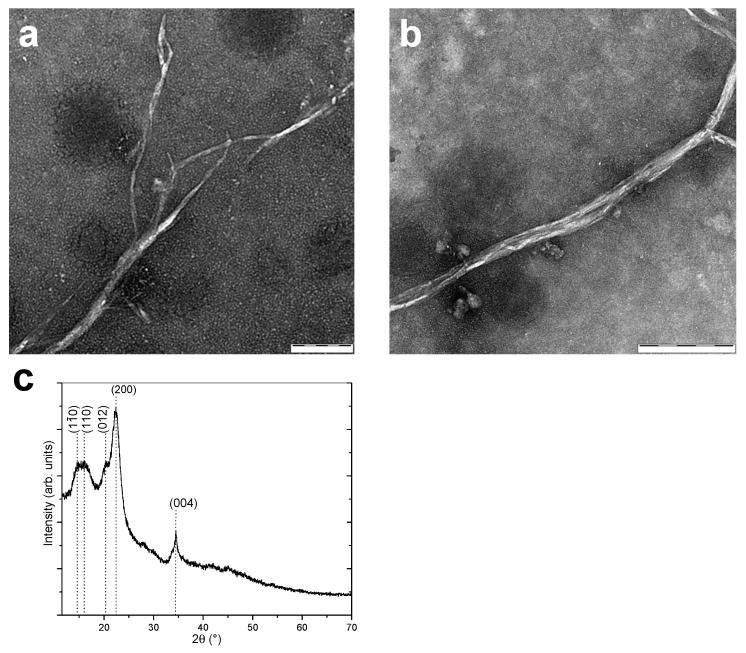
(**a**,**b**) TEM images of cellulose nanofibers (CNFs) at different magnifications. Scale bars: (**a**) 100 nm; (**b**) 200 nm. (**c**) Wide angle X-ray diffraction pattern of the CNFs, with indexed crystallographic planes of native cellulose I allomorph.

Figure 1 shows TEM micrographs and the X-ray diffraction pattern of the cellulose nanofibers (CNF) used to produce the CNF-filled chitosan inks and printed scaffolds. The CNFs consisted of an entangled network of interconnected nanofibrils with very high aspect ratio (length/width), with an average width of 35.2 ± 8.1 nm and bundles of up to around 50 nm width. The relatively high value of this latter could be related to the drying during TEM sample preparation, which might induce partial fibril aggregation [74]. The mechanoenzymatic hydrolysis used to produce the nanofibers yields long nanofibrils preserving the native cellulose I crystalline allomorph structure, as displayed in the X-ray diffraction pattern (Figure 1c), with partly amorphous regions, which are able to inherently entangle and form a fibril network with hairy branches [74]. The preserved native cellulose I allomorph and the intramolecular hydrogen bonding within amorphous and crystalline phases can lead to efficient nanoreinforcement effect, above from fibers consisting of regenerated cellulose II allomorph.

### 3.2. Chitosan/Cellulose Nanofiber Inks Rheological Behavior

**Figure 2:**
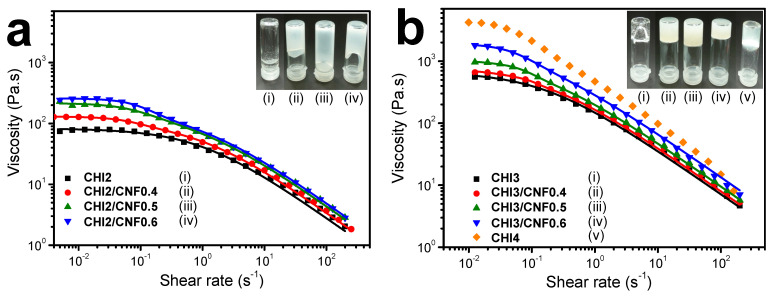
Flow diagrams of the CNF-filled CHI viscous inks (**a**) and the corresponding naked CHI inks (**b**). Solid lines represent the fitting of the rheological behavior with the Cross model equation (Equation (1)) for CHI solutions, and with the double Cross model equation (Equation (2)) for the CHI/CNF suspensions. Insets in show pictures of the different viscous inks.

Figure 2 shows the flow diagrams of the CNF-filled chitosan viscous suspensions and the corresponding chitosan solutions. A steady plateau corresponding to the Newtonian viscosity is observed for all characterized samples. Two distinct regions can be observed in the different curves: At low shear rates, the Newtonian flow region shows a constant zero-shear viscosity (η_0_), as the provided shear forces to disentangle the polymer chains appear to be lower or equal to those maintaining them tangled; at higher shear rates, a shear-thinning behavior is observed with a decrease of viscosity as the shear rate increases [77,78].

The flow diagrams (*η* vs. $\dot{\gamma }$ = d*γ*/d*t*) of the “pure” chitosan solutions (Figure 2) could be modelled with the three-parameter Cross law (Equation (1)): [79,80,81]

(1) $$\eta =\frac{\eta_{0,CHI}}{1+{\left(\dot{\gamma }\tau_{CHI}\right)}^{1-n_{CHI}}}$$

The Cross equation yields the Newtonian or zero-shear viscosity *η*_0_, the flow behavior index *n*, and the relaxation time of chitosan polymer chains *τ*, as displayed in Table 2. As expected, the steady-state shear viscosity of the pure CHI inks increased with increasing CHI concentration. The polymer chain interactions and entanglements increased with the polymer concentration, restricting the chain relaxation into disentanglement. The CHI/CNF viscous inks exhibited more complex flow diagrams, with higher Newtonian viscosities measured in the low shear rate range ($\dot{\gamma }$ < 1 s^−1^) and shear thinning occurring in two different regimes, which was more evident for higher CNF contents (Figure 2). Thus, these two-step flow diagrams of the CHI/CNF suspensions could be modelled with a double Cross law (Equation (2)):

(2) $$\eta =s\frac{\eta_{0,CHI}}{1+{\left(\dot{\gamma }\tau_{CHI}\right)}^{p_{CHI}}}+\frac{\eta_{0,CNF}}{1+{\left(\dot{\gamma }\tau_{CNF}\right)}^{p_{CNF}}}$$

where *η*_0,*CNF*_, *τ_CNF_*, and *p_CNF_* = 1− *n_CNF_* are the flow parameters of CHI chains, possibly due to chains interacting with CNFs in the CHI/CNF suspensions in the slower flow regime. Rheological model fitting used a Levenberg–Marquardt nonlinear regression algorithm in the Octave 4.4.0 programming environment [22]. Table 2 shows the flow parameters obtained for the CHI/CNF suspensions and the Newtonian viscosity *η*_0,*CHI*_, the relaxation time *τ_CHI_*, and the exponent *p_CHI_* = 1 − *n_CHI_* for pure CHI solutions. For a given chitosan concentration, the presence of CNF increased the Newtonian viscosity measured at low shear rates. With the increase of the shear rate, the viscosity of both the pure CHI viscous solutions and the CHI/CNF inks similarly decreased, revealing almost the same shear-thinning behavior. In the CHI/CNF formulations two different chain relaxation phenomena might occur. In the “pure” CHI inks, the main chain relaxation, being dominating at high shear rate and corresponding to the disentanglement of the polymer chains transient network, shows relaxation time of 1 s in inks with 2% (*w/v*) CHI, and around 6 s in inks with 3% (*w/v*) CHI (Table 2) [82,83]. At a low shear rate, in the presence of CNFs, a second relaxation occurs with relaxation times of the order of 10 s (practically independently of the CNF content, except for the formulation CHI3/CNF0.6 with the highest CHI and CNF concentrations). To conclude, the higher Newtonian viscosity measured at low shear rates in the CHI solutions filled with CNFs, in comparison to the pure CHI solutions, and their similar shear-thinning behaviors with similar flow exponents at high shear rates, could be explained due to the CHI polymer chain relaxation (disentanglements) impacted by the CHI concentration [84]. As mentioned above, the CNFs surface is weakly charged with carboxylate moieties displaying a surface charge density of 40–80 mmol/kg [85,86]. Weak electrostatic interactions could establish between the CHI polycation and the CNF polyanionic surface, allowing for stress transfer from the CHI solution matrix to the nanofibers. According to the results, in the CHI/CNF inks the establishment of a rigid cellulose network with permanent CNF–CNF interactions would not occur, as a gel-like flow behavior with *η*~1/$\dot{\gamma }$ would be instead observed [87]. Actually, CHI chains could absorb on the CNFs surface and play a role in the bridging of nanofibers [88,89], resulting in entanglements formation between the adsorbed chains and the other chains in the solution. Addition of the CNFs, even at low concentration, is likely to impact the dynamics of CHI chains since the surface area of the nanofibers is very large [58].

Thus, the relaxation time *τ* for chitosan is hypothesized to be interdependent with the incorporation of CNF. When adding the CNFs, the observed relaxation time *τ_CNF_* can increase due to both the increase of CNF content and of CHI concentration (Table 2). Such relaxation time is tentatively related to the rupture of CNF/CHI network (CNF strands bridged by chitosan chains), which effect seems to be more important after reaching a threshold of CHI concentration beyond 2% (*w/v*). Increasing the CNF content should result in a denser CNF network, possibly involving rearrangements at larger scales of CNF aggregates. Then, higher CHI concentration also should contribute to the CNF/CHI network, since adsorbed CHI chain would act as fibril binders. It also will imply a denser network with longer relaxation times, associated with larger scale reorientation and interfibrillar bridge rupture with applied strains. Then, the decrease of the exponent *p_CNF_* (Table 2) consistently should reflect a more heterogeneous CNF network disruption process at higher CNF contents, specially emphasized in systems with higher CHI concentration. Nevertheless, the study of the flow diagrams in the extended shear rate range, for example by capillary rheometry with use of the Weissenberg–Rabinowitsch correction as we previously investigated (Doench et al. [22]), would be necessary to determine the exponent parameter *p* with precision.

Thus, the CHI/CNF inks flow at high shear rates is dominated by the CHI chains disentanglement and is less affected by the presence of the CNFs. This is advantageous for printability by extrusion of this system, as CNFs can contribute to improve mechanical properties and increase the zero shear viscosity while practically not affecting the extrudability of CHI-based systems (higher shear rates).

### 3.3. Cellulose Nanofiber-Filled Chitosan Printed Hydrogels

#### 3.3.1. Morphology and Dimensions

**Figure 3:**
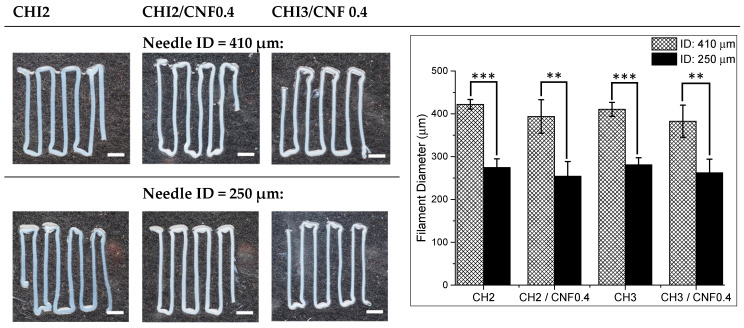
Photos of the hydrogel filaments obtained by microextruding CHI/CNF viscous suspensions of different compositions through needles with tip inner diameters (ID) of 250 or 410 μm (scale bars: 1500 μm). Histograms with hydrogel filament mean diameters obtained after printing the different CHI/CNF formulations. Right: Printed hydrogel average filament diameters obtained for the different CHI/CNF formulations, using the two different extrusion needle ID. For the statistical analysis, independent sample *t*-tests were performed with STATISTICA 10.0 (StatSoft Inc: Tulsa, OK, USA, 2011). *p* < 0.05 was accepted as statistically significant. Filament diameter values are expressed as means ± SDs. *n* = 6 (** *p* < 0.01, *** *p* < 0.001).

Figure 3 shows images of hydrogel filaments printed from different CHI/CNF formulations by varying the extrusion needle inner diameter (ID) and pressure as displayed in Table 1. The addition of CNFs into the CHI viscous solutions slightly decreases the diameter of the printed hydrogel filaments. This might be related to a limited swelling at the die outlet due to the increased viscosity and relaxation time resulting from mild interaction between CHI and CNFs, as inferred from the rheological analysis. In general, the decrease of extrusion needle inner diameter offers an increase resolution of printed filaments [90]. Thus, the combined effect of the composition of the CHI/CNF formulation, needle inner diameter, and pressure contribute to the size resolution of the hydrogel filaments.

**Figure 4:**
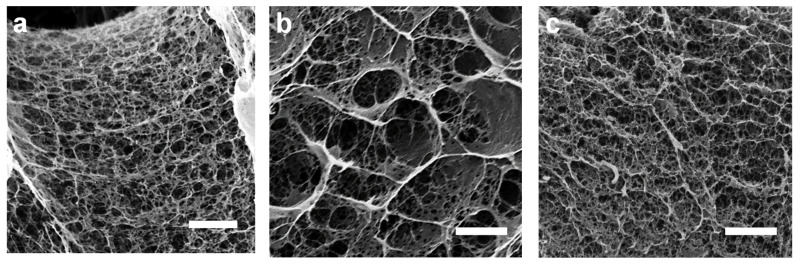
Scanning electron micrographs (SEM) of hydrogels printed from neat chitosan CHI 2% and 3% (*w/v*) ink formulations ((**a**,**c**), respectively), and from CHI2/CNF0.4 ink formulation (**b**). Scale bars: 5 µm.

Figure 4 shows scanning electron microscopy (SEM) micrographs of the cross-section of the CHI/CNF filaments after freeze-drying. Although the lyophilizates are known to exhibit a coarser morphology in comparison with hydrogels [91], the obtained scaffolds show a fine spider web-like network microstructure with interconnected fibrils, which might facilitate cell adhesion, proliferation, and migration. Cellulose nanofibers were not distinguishable, which should confirm a good dispersion of the CNFs in the composite. The SEM images revealed that with the increase in CHI concentration, the porosity decreases and the pore sizes strongly increases with the incorporation of CNFs (Figure 4). In one hand, the nanofibers seem to favor microstructures with more expanded flat walls with still residual fibrillar network morphology (Figure 4b). Sereni et al. [92] reported on the formation of radial capillaries or microrange tubular pores in CHI physical hydrogels, related to directional neutralization of chitosan chains during hydrogel processing. The addition of CNF has influence on CHI/CNF suspension viscosity, which should impact the size of resulting tubular pores in hydrogels [92]. Such tubular porosity revealed in related biopolymer hydrogels might be also observed in the freeze-dried scaffolds.

#### 3.3.2. Mechanical Properties

**Figure 5:**
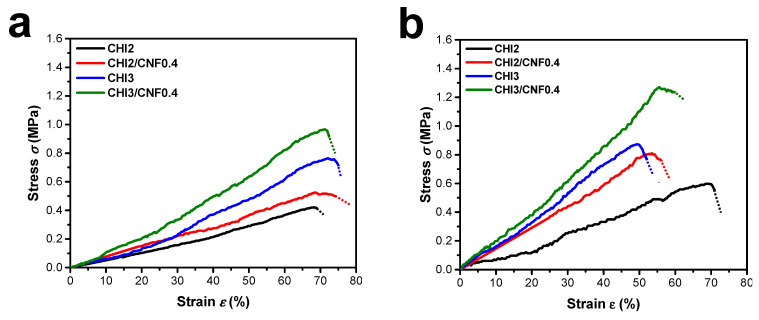

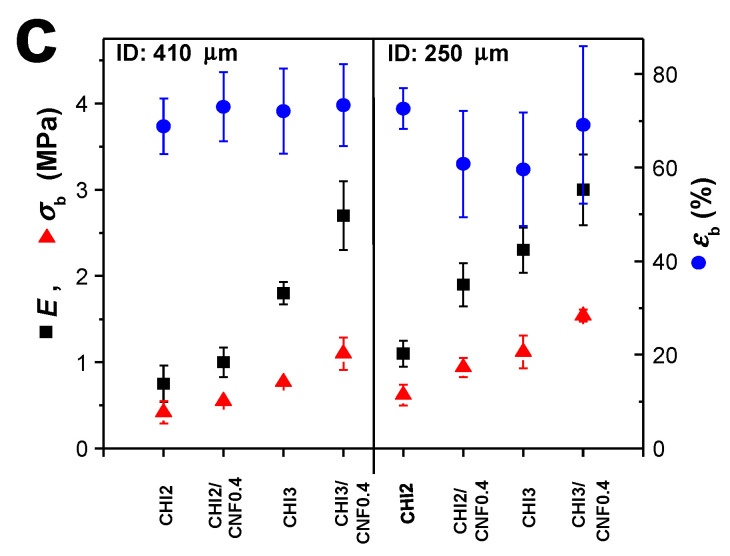
Stress–strain curves of uniaxially stretched printed hydrogel filaments of chitosan (CHI) alone and cellulose nanofiber (CNF) filled chitosan composites, for different CHI and CNF concentrations, by using conic extrusion needles with inner diameter (ID) of: (**a**) 410 μm and (**b**) 250 μm. (**c**) Young’s modulus (E), stress (σ_b_), and strain at break (ε_b_) achieved for the tensile tested filaments in (**a**,**b**), obtained with the two different extrusion needle IDs.

Figure 5 shows the stress–strain curves of microtensile testing performed on the printed CHI and CHI/CNF hydrogel filaments. Figure 5c summarizes the achieved Young’s modulus (E), stress (σ_b_), and strain at break (ε_b_) obtained for filaments of different compositions and printing conditions. The mechanical behavior was influenced by the CHI concentration, the CNFs content and the printing processing parameters like the extrusion needle inner diameter (ID). The Young’s modulus and tensile strength increased with the CHI and CNF concentrations, but decreased with the increase of needle inner diameter. As expected, varying CNFs concentrations from 0 to 0.4% induced a significant enhancement in the mechanical properties of composite hydrogel filaments. Finally, mechanical properties of CHI hydrogels could be improved by reinforcing with the CNFs. The enhanced properties are due to the CNFs nanoscale (large specific surface area), high Young’s modulus, and aspect ratio typical of nanofibrillated cellulose [21,22,42,43,56]. In the hydrogel composites, an efficient matrix/reinforcement interaction should contribute to the stress transfer from the CHI matrix to the nanofibers, thereby yielding higher stiffness and strength. Moreover, in the microtensile testing the uniaxial stretching was performed in the same direction as the axis of the extrusion needle. Thus, the obtained mechanical properties should be also impacted by the orientation of both the CHI polymer chains and the CNFs along the hydrogel extrudate axis, due to shear-induced orientation during extrusion. Such effect could be more significant when using a thinner extrusion needle like that of ID = 250 μm (Figure 5).

#### 3.3.3. Synchrotron X-ray Scattering SAXS/WAXS Analyses of Printed Hydrogels. Characterization of the Cellulose Nanofibers Dispersion and Orientation

**Figure 6:**
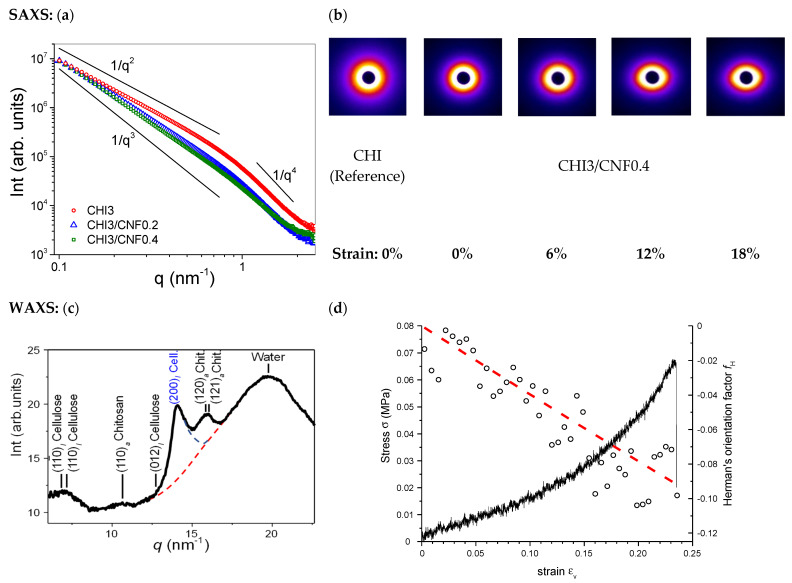
(**a**) Synchrotron small-angle X-ray scattering (SAXS) radial average curves of the CHI/CNF printed hydrogel filaments; (**b**) 2D SAXS image patterns recorded for different strains after stretching the CHI3/CNF0.4 printed hydrogel formulation, with printed CHI hydrogel reference included. (**c**) Synchrotron wide-angle X-ray scattering (WAXS) radial average of printed CHI3/CNF0.4 hydrogel composite, for example, after 14% of tensile strain; (**d**) Evolution of the Herman’s orientation factor *f*_H_ estimated from the azimuthal intensity plots of the (200)_I_ reflection of cellulose I (fitted with Lorentz function) at different strain values during in situ uniaxial stretching (rate: 1 µm/s, RH: 45%) using X-ray synchrotron radiation, for the CHI3/CNF0.4 printed hydrogel. The red dashed-line in (**d**) shows the CNF orientation distribution function obtained by the affine model used for reorientation of rigid rod-like crystals [95,96].

Microstructural characterization was also performed by synchrotron X-ray scattering SAXS and WAXS of the CHI/CNF printed hydrogel filaments. Figure 6a shows the SAXS analyses of printed CHI/CNF hydrogel composites and the corresponding CHI reference. In the low q-range (where qR_0_ << 1, with R_0_ being the radius of nanofibrillar objects), a scattering law between 1/q^2^ and 1/q^3^ was observed, closer to 1/q^2^ for the pure CHI hydrogel, and to 1/q^3^ for the CHI/CNF systems. This scattering laws could be attributed to a large distribution of cross-section radii R_0_ of the fibril-like features constituting the hydrogel fibrillar network microstructure in pure CHI hydrogels [40,93], as revealed by SEM (Figure 4); and also to the morphology of the CNF rod-like particles with broad cross-sectional size distribution. In the q-range for *q* > 0.7 nm^−1^, the Porod’s law (I(q) = C/q^4^) is evidenced, revealing the sharp electron density variation within the microstructure. The Porod’s law can be used to estimate the Porod’s length ($l_{p}=\frac{V_{CNF}}{S_{CNF}}\sim \frac{Q}{\varphi_{CNF}\pi C}$) of the CNFs, where *Q* is the scattering invariant $Q=\overset{\infty }{\underset{0}{\int }}I\left(q\right)q^{2}dq$, and C the Porod’s constant [94]. Then, the invariant *Q* can be estimated using the Equation (3), which is divided in three subintegrals (*Q*1, *Q*2, and *Q*3) corresponding to integration in different regions of the SAXS scattering curve (Figure 6a): (*Q*1) at the lowest (low-*q* Guinier region, *q*→0), (*Q*2) middle (intermediate-*q* Guinier region) with *q_max_* around 0.7 nm^−1^, and (*Q*3) final slope (Porod region) *q*-values, with the lowest *q*-angle SAXS trend of the regions (1) and (i2) mainly obtained by extrapolation.

(3) $$Q=\overset{\infty }{\underset{0}{\int }}I\left(q\right)q^{2}dq=\overset{q_{min}}{\underset{0}{\int }}Aq^{2+\alpha }dq+\overset{q_{max}}{\underset{q_{min}}{\sum }}I\left(q_{i}\right)q_{i}^{2}\Delta q+\overset{\infty }{\underset{q_{max}}{\int }}\frac{C\cdot \exp\left(-s^{2}q^{2}\right)}{q^{2}}dq$$

The Porod’s constant *C* was deduced by non-linear least square fit (using lsqcurvefit() function in Matlab). The approximate value of the Porod’s length *l_p_* was expressed as $l_{p}\sim \frac{Q}{\pi C}$, with *Q* being the sum of the subintegrals *Q*_1_, *Q*_2_, and *Q*_3_. For example, from the SAXS curve of the formulation CHI3/CNF0.4, containing 3% (*w/v*) CHI and 0.4% (*w/v*) CNF, the resulted *l_p_* value was ~8 nm, which should correspond to the smallest width of the cellulose fibrils within the suspension. This fairly agrees with the width range of the individualized cellulose nanofibrils as observed by TEM (Figure 1a), demonstrating that an excellent dispersion of the CNFs was obtained within the printed CNF-filled chitosan hydrogel composites.

Then, uniaxial stretching of the printed hydrogel filaments was performed at the synchrotron beamline to allow in situ microstructural characterization by X-ray scattering (SAXS/WAXS) and the orientation of the CNFs within the CHI/CNF hydrogels and its quantification (Figure 6a). The appearance of the SAXS patterns in Figure 6b (Right), which become more anisotropic when the strain values increased, shows that the preferential orientation of CNF and chitosan nanofibrils in the hydrogel biomaterial could be further increased at stretching the printed filaments. From the 2D WAXS synchrotron patterns of the CHI/CNF hydrogels, the Herman’s orientation factor *f_H_* of cellulose crystals could be calculated. To this end, the 2D X-ray scattering images were azimuthally sectorized, and the intensity peak around the (200)_I_ crystallographic ring of Cellulose I allomorph was deconvoluted (Figure 6c) for each sector centered at the azimuthal angle *φ_k_*. This calculation was performed for different diffraction images obtained for different strain values (Figure 6d) during stretching the CHI/CNF hydrogel filament at the synchrotron X-ray beamline. From this *I*_200,k_ sectorization, the Hermans’ orientation factor for the (200)_I_ reflection of Cellulose I was obtained as follows (Equation (4)):

(4) $$f_{H}=\frac{\int_{\varphi =0}^{360}I_{200}(\varphi )cos^{2}(\varphi )\sin(\varphi )d\varphi }{\int_{\varphi =0}^{360}I_{200}(\varphi )\sin(\varphi )d\varphi }=\frac{\sum_{k\sec tor}I_{200,k}cos^{2}(\varphi_{k})\sin(\varphi_{k})\Delta \varphi }{\sum_{k\sec tor}I_{200,}{}_{k}\sin(\varphi_{k})\Delta \varphi }$$

where Δ*φ* is the azimuthal angle differentiate for each sector. The details of the deconvolution fit with Lorentz functions and parabolic contribution of water, the treatment of the sector-averaged azimuthal intensity vs. azimuthal angle *φ_k_*, and the resulting calculation of the <cos^2^*φ*> are described in the Supporting Information (Supplementary Materials Figure S1 and Equation (S2)). Figure 6d shows that the Hermans’ orientation factor evolved from close to 0 (random orientation) to −0.11 (anisotropic orientation of CNF) at stretching the CHI3/CNF0.4 printed hydrogel till strain around 20%. For printed CHI3/CNF0.2 hydrogels, a similar decrease of *f_H_* until down to −0.08 was obtained after a 20% applied strain (data not shown). The evolution of the *f_H_* values confirms the enhancement of alignment of CNFs in the stretching axis, which enhances the mechanical performance of the hydrogel composites. The calculated values of Hermans’ factor *f_H_* due to CNF orientation are in good agreement with the affine model calculation [95,96] shown in Figure 6d (red) (see also Supplementary Materials Figure S1), describing the reorientation of rigid rod-like crystals.

#### 3.3.4. Experimental Design for the Optimization of the Size Resolution and Mechanical Performance of CHI/CNF Printed Hydrogels

**Figure 7:**
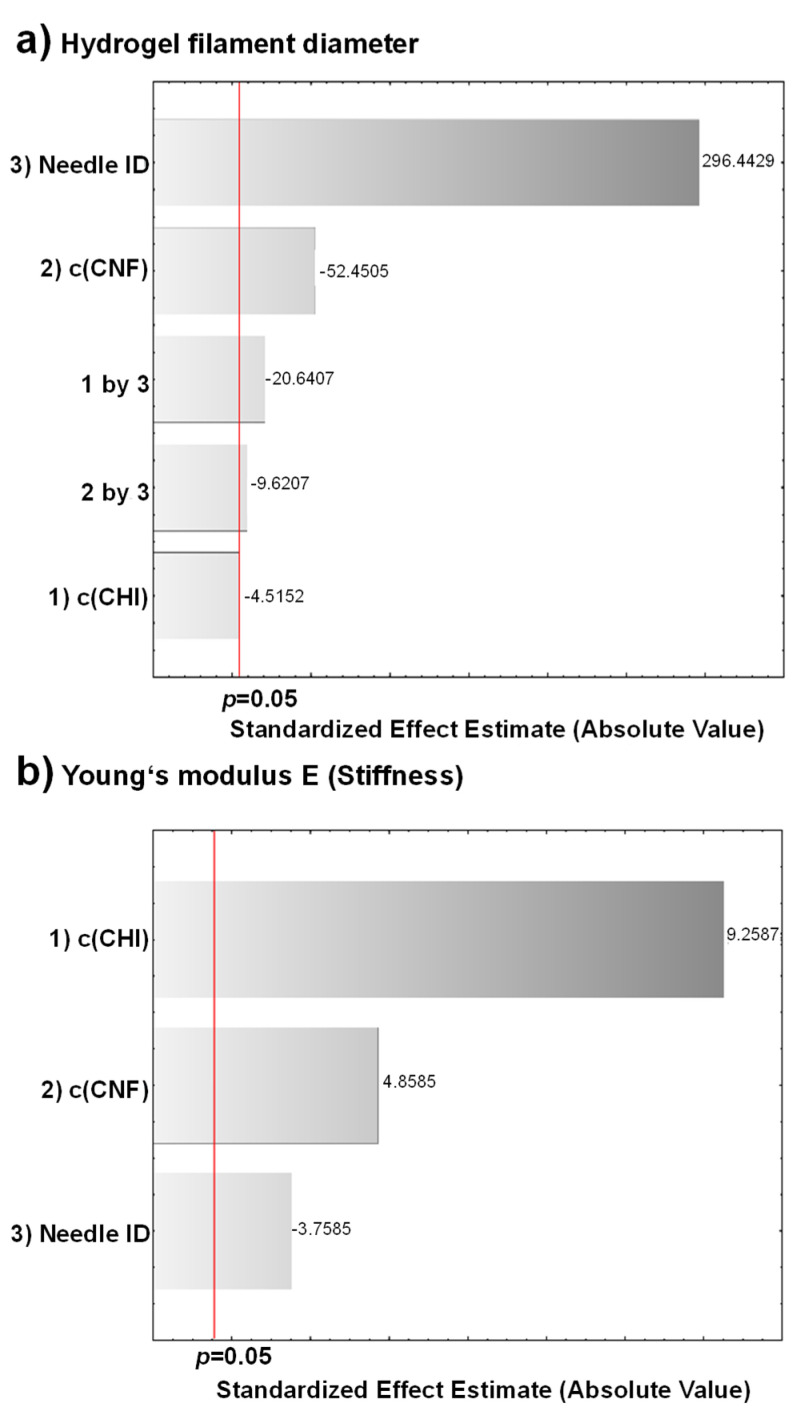

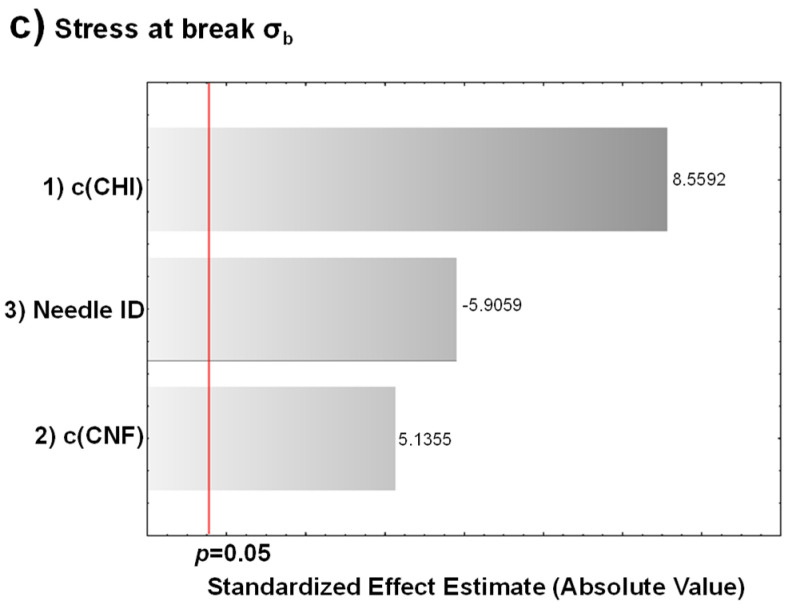
Pareto chart of the three responses: (**a**) hydrogel filament diameter, (**b**) Young’s modulus E, and (**c**) stress at break σ_b_, after considering a two level variation of the variables: (1) c(CHI), (2) c(CNF), and (3) needle ID, in a 2^3^ factorial experimental design.

An experimental design allowed statistically evidencing the impact of the processing parameters discussed above on the printed hydrogel filament diameter and mechanical properties. To determine the most significant parameters affecting the resolution and mechanical performance of the printed CHI/CNF hydrogels, the three independent variables: concentration of chitosan c(CHI), cellulose nanofibers content c(CNF), and printing extrusion needle tip inner diameter (ID) were considered in the statistical analysis. As above, the c(CHI), c(CNF), and needle ID were varied following two levels: c(CHI): 2% and 3% (*w/v*); c(CNF): 0% and 0.4% (*w/w*); needle ID: 250 and 410 μm. A 2^3^ factorial design was used, including the eight formulations described in Table 1. The run order of the experiments was randomized to prevent systematic errors. The following responses: printed CHI/CNF hydrogel filament diameter (Figure 3) and mechanical properties (Young’s modulus E, and stress at break σ_b_) (Figure 5) were evaluated with the analysis of variance (ANOVA) using the software STATISTICA 10.0 (StatSoft Inc: Tulsa, OK, USA, 2011). The significance of the effects was verified with Fisher’s statistical test using 0.05 as significance level. ANOVA analyses showed that the CNFs content and the needle ID were the most significant variables (*p* < 0.05) influencing on the diameter of the printed hydrogel filaments. Concerning mechanical properties, the three factors: (1) c(CHI); (2) c(CNF); (3) needle ID were significant for the Young’s modulus and the stress at break of the printed filaments. Interactions of c(CHI)*needle ID (1 by 3) and of c(CNF)*needle ID (2 by 3) also have significant influence on the filament diameter. The ANOVA results are shown in Supplementary Materials Tables S1–S3 while Pareto Charts are depicted in Figure 7. The needle ID has the strongest effect on the hydrogel filament diameter. As expected, a thinner needle leads to smaller filament diameters. The c(CNF) has a negative effect on this response, which means that higher c(CNF) is needed to achieve thinner filaments. The interaction between c(CHI) and needle ID (1 by 3) also has a negative effect on the filament diameter. When the needle ID is in its lower level (250 μm), the c(CHI) also should be in its lower level (2% (*w/v*)) in order to reach the lowest filament diameter. Similarly, if the needle ID is in its higher level (410 μm), a high c(CHI) is preferred to develop filaments with smaller diameter. These effects are shown in Supplementary Materials Figure S2. For the mechanical properties, the c(CHI) and c(CNF) have positive effect, while needle ID has a negative one. Thus, higher Young’s modulus and stress at break are achieved for higher concentrations of CHI, CNF, and smaller needle ID. Finally, as both responses are important to achieve printable and functional CHI/CNF hydrogel filaments of high size resolution and good mechanical properties, which might be a premise for the 3D printing of hydrogel constructs, the desirability function was used to simultaneously optimize the two responses (filament diameter and mechanical properties). This function is based on a numerical interval that defines the desirability of the analyst in relation to the process optimal condition. To achieve hydrogel filaments with the lowest diameter and the highest mechanical performance, the optimization using the desirability function yielded as optimum processing parameters: c(CHI) = 3% (*w/v*); c(CNF) = 0.4% (*w/w*); needle ID = 250 μm (Supplementary Materials Figure S3), corresponding to that used in the formulation F8 of Table 1.

### 3.4. 3D Printed CHI/CNF Hydrogel Scaffolds

**Figure 8:**
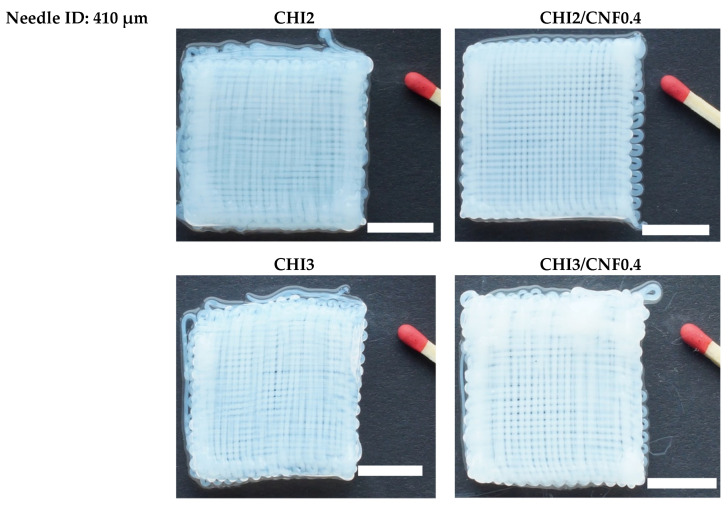

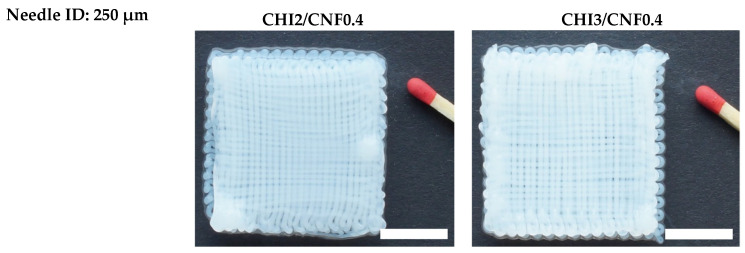
The 3D printed scaffolds of chitosan/cellulose nanofiber hydrogels with 30 × 30 mm^2^ and 0.85 mm interfilament distance. Scale bars: 11.5 mm.

The obtained good conditions for hydrogel filament resolution and mechanical properties were considered for the printing of hydrogel scaffolds and in vitro cell culture experiments. The 3D scaffolds of varied CHI and CNF concentrations were printed for comparison, and with the different needle IDs, to demonstrate the feasibility of extending the above results into printing approaches in 3D, in the form of layered hydrogels. Again, the benefit of adding CNFs as reinforcement in CHI hydrogels to print 3D CHI/CNF hydrogel composites was clearly observed, where 3D CHI-based scaffolds of both 2% and 3% (*w/v*) chitosan containing CNFs amount as low as 0.4% showed lower filament diameter, i.e., better resolution than those scaffolds only containing chitosan (Figure 8).

#### Suitability of 3D Printed CHI/CNF Hydrogel Scaffolds for Three-Dimensional Cell Culture

**Figure 9:**
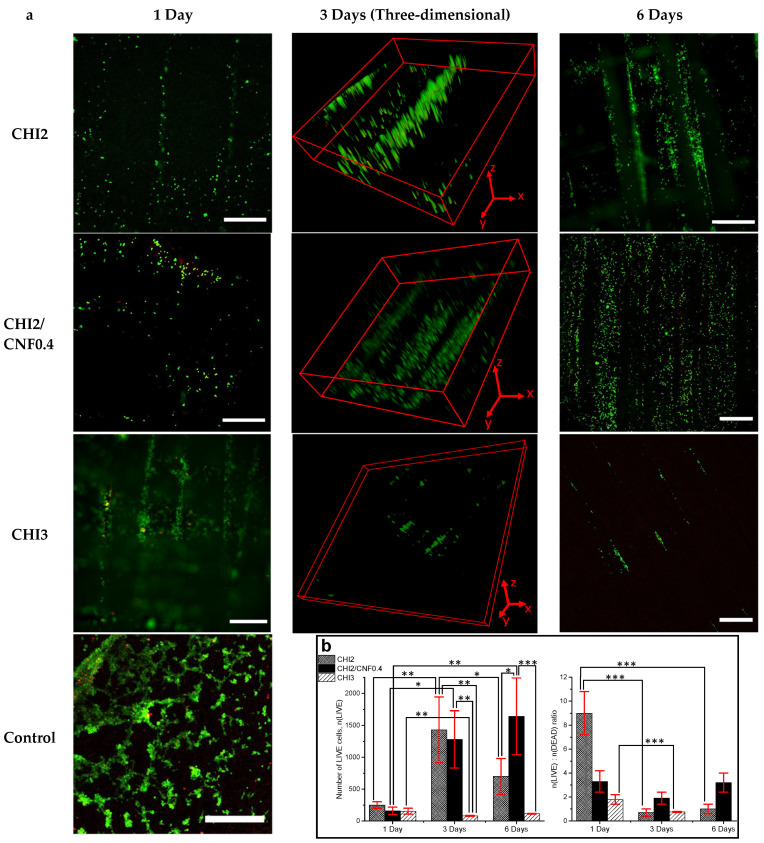
(**a**) LIVE/DEAD assay confocal laser scanning microscopy (CLSM) images after culture of NIH/3T3 fibroblast cells in 3D printed CHI/CNF hydrogel composite scaffolds of different CHI and CNF concentrations (Figure 8). LIVE indicator: green fluorescence; DEAD indicator: red fluorescence. Scale bars: 500 μm. Three-dimensional frames (3 days) scale: CHI2) x*y*z: 2.3*2.3*0.075 mm^3^; CHI2/CNF0.4) x*y*z: 2.3*2.3*0.50 mm^3^; CHI3) x*y*z: 3.0*3.0*0.15 mm^3^. (**b**) **(Left)** Number of LIVE cells *n*(LIVE); (**Right**) *n*(LIVE)/*n*(DEAD) cell ratio obtained for the different CHI/CNF formulations at the different days, expressed as means ± SDs, *n* = 4 (* *p* < 0.05, ** *p* < 0.01, *** *p* < 0.001).

NIH/3T3 fibroblast cells were cultured in the printed multilayer CHI/CNF hydrogels. Three different ink formulations (CHI2, CHI3, and CHI2/CNF0.4) were considered in the cell culture studies. LIVE/DEAD cell viability assays at Day 1, 3, and 6 were performed. Figure 9a shows micrographs obtained at the confocal fluorescent microscope for different scaffold compositions at the corresponding culture times.

Cells proliferated within the accessible interfilament spaces predefined for the printed multilayer hydrogel mesh, which yielded cellularized bioconstructs. For the lower CHI concentration (2% (*w/v*)), after initial cell adhesion on the filaments surface, the cells also colonized the inner microstructure of the hydrogel filaments as clearly observed after days 3 and 6 for the CHI2 and for the CHI2/CNF0.4 formulations (Figure 9). This was hardly observed in the mesh printed with CHI 3% (*w/v*), which might be related to a lower porosity of the hydrogel filaments at higher CHI concentrations (as displayed in the SEM observation of corresponding lyophilizates, Figure 4c), which might hinder cell colonization in the denser hydrogel. This led to significant cell death after 6 days of incubation in the CHI3 formulation. In contrast, for scaffolds prepared with CHI 2% (*w/v*), which in addition contained 0.4% of CNFs, an appreciable colonization of cells was observed both in the inner porous microstructure of the CHI2/CNF0.4 hydrogel filaments and in the interspaces (big pores) of the scaffold mesh with dimensions predetermined in the 3D printing process. Thus, fluorescent micrographs after 3 and 6 days of culture in the CHI2/CNF0.4 constructs display a growing of cells in 3D, which becomes more significant and homogeneous after 6 days (Figure 9). The incorporation of CNFs at that low content of 0.4% (*w/v*), in hydrogels prepared with low CHI concentration like 2% (*w/v*), seems to phenomenologically yield the appropriate composition for the best in vitro response, which related microstructure is displayed in the SEM analysis of Figure 4b in comparison to the other compositions. The achieved pore sizes combination, the inner walls surface of the composite microstructure and the mechanical properties (Young’s modulus: 2 MPa, stress at break: 1 MPa) of this CHI2/CNF0.4 composition seems to be optimal for cell adhesion and proliferation with guaranteed nutrients exchange in the 3D hydrogel scaffold, resulting in homogeneous cell colonization within the whole scaffold (Figure 9). Thus, the addition of CNFs to CHI improves hydrogel stiffness and does not compromise the biocompatibility of chitosan and viability of cells.

The above findings were quantitatively evaluated and demonstrated in the statistical analysis of the LIVE and DEAD counted cells (see also Supporting Information). Figure 9b reports the number of LIVE cells counted after 1, 3, and 6 days of culture of fibroblasts within the 3D printed hydrogel scaffolds containing different concentrations of CHI and CNFs. Between the different formulations, after 3 days significant differences were observed in the number of living cells with more alive cells in the formulations containing low chitosan concentration like the CHI2 and CHI2/CNF0.4, respect to the CHI3 hydrogel scaffold; and with no significant differences in the number of living cells between those formulations of low chitosan concentration (CHI2 and CHI2/CNF04). Then, after 6 days incubation, the number of living cells in the cellulose nanofiber-filled chitosan hydrogel was significantly higher than in the scaffolds prepared with chitosan alone (Figure 9b). The ratio of living to dead cells n(LIVE): n(DEAD) was also statistically evaluated (Figure 9b) and significant differences were observed in the CHI2 and CHI3 formulations for the days 3 and 6 with respect to day 1, this is with a significant increase of the number of dead cells after day 3. In contrast, in the formulation containing the cellulose nanofibers (CHI2/CNF0.4) the balance between LIVE and DEAD cells did not significantly change along the considered culture days.

## 4. Conclusions

The engineering of purely natural and mechanically performant bioinspired 3D printed hydrogels, for application in tissue engineering, of chitosan (CHI) hydrogels filled with cellulose nanofibers (CNFs) without any modification of the biopolymer constituents and addition of any chemical crosslinker, was achieved by extrusion-based 3D printing technology. The addition of cellulose nanofibers with high mechanical properties into chitosan hydrogel ensured good printing ability and printed constructs resolution without compromising chitosan bioactivity and biocompatibility. The viscosity of the printable CHI/CNF suspensions (inks) was as low as 100–500 Pa·s at shear rate 1 s^−1^ and allowed the deposition of gel filaments of good mechanical performance and printing resolution (220–430 μm). Stable 3D hydrogel meshes were obtained with very low concentration both of the biopolymer matrix and the nanofiber filler, still supporting three-dimensional cell colonization and good cell viability, yielding cellularized bioconstructs. Finally, the most relevant CHI/CNF biomaterial technical characteristics were optimized to produce natural bioinspired 3D functional fiber-filled hydrogels for tissue engineering applications with great potential in the repair of mechanically demanding hydrogel tissues like intervertebral disc, cartilage, meniscus, among others. The immediate perspective of this work is the development of different 3D-shape bioconstructs, targeting the anisotropic and multilamellar hydrogel structure of intervertebral disc regions. This will constitute a forward step of our previous studies dedicated to assess biocompatible and functional composite hydrogel implants in disc tissue engineering and regeneration, with the advantage of using 3D printed more-mimicking hydrogel environments for cell and tissue growth.

## 5. Patents

Osorio-Madrazo, A.; David, L.; Montembault, A.; Viguier, E.; Cachon, T. Hydrogel Composites Comprising Chitosan and Cellulose Nanofibers. International Patent Application No. WO 2019/175279 A1, 19 September 2019; US Patent App. 16/980,383, 12 February 2021.

## Figures and Tables

**Table 1:** Processing parameters for the printing of hydrogel filaments and 3D hydrogel scaffolds of CHI/CNF formulations.

Formulation	CHI % (*w/v*)	CNFs % (*w/w*)	Needle Inner Diameter ID (μm)	Extrusion Pressure (bar)
F1 (CHI2)	2	0	410	0.15
F2 (CHI2/CNF0.4)	2	0.4	410	0.25
F3 (CHI3)	3	0	410	0.35
F4 (CHI3/CNF0.4)	3	0.4	410	0.47
F5 (CHI2)	2	0	250	0.25
F6 (CHI2/CNF0.4)	2	0.4	250	0.35
F7 (CHI3)	3	0	250	0.70
F8 (CHI3/CNF0.4)	3	0.4	250	0.77

**Table 2:** Flow parameters determined from the viscosity vs. shear rate curves (Figure 2) of different CHI/CNF compositions, by using the Cross model (Equation (1)) for naked CHI inks and double Cross model (Equation (2)) for CHI/CNF formulations.

Formulation	*η*_0*,CHI*_ (Pa·s)	*τ_CHI_* (s)	*p_CHI_*	*s*	*η*_0,*CNF*_ (Pa·s)	*τ_CNF_* (s)	*p_CNF_*
CHI2	83	1.0	0.73	-	-	-	-
CHI2/CNF0.4	83	1.0	0.73	1.22	30.4	11.9	1.96
CHI2/CNF0.5	83	1.0	0.73	1.56	87.8	9.6	1.46
CHI2/CNF0.6	83	1.0	0.73	1.75	116.3	8.6	1.95
CHI3	674	6.0	0.71	-	-	-	-
CHI3/CNF0.4	674	6.0	0.71	1.11	39.3	8.7	6.55
CHI3/CNF0.5	674	6.0	0.71	1.33	207.3	10.9	2.87
CHI3/CNF0.6	674	6.0	0.71	1.88	752.1	13.9	1.89

## Data Availability

Not applicable.

## Supplementary Materials

The following are available online at https://www.mdpi.com/article/10.3390/polym13101663/s1, Figure S1: Azimuthal intensity around the diffraction signal (200)*_I_* of cellulose I crystals constituting the cellulose nanofibers (CNFs) vs. azimuthal angle φ; Figure S2: Effect of the interaction between c(CHI) and needle ID on the hydrogel filament diameter; Figure S3: Profiles of predicted values and desirability; Table S1: ANOVA analysis for printed hydrogel filament diameter, Table S2: ANOVA analysis for Young’s modulus E of printed hydrogel filament, Table S3: ANOVA analysis for stress at break of printed hydrogel filament.
