# Supplementary material for: Development of Bioinspired Functional Chitosan/Cellulose Nanofiber 3D Hydrogel Constructs by 3D Printing for Application in the Engineering of Mechanically Demanding Tissues
Source: Polymers (Basel). 2021 May 20;13(10):1663. doi: 10.3390/polym13101663 (PMC8160918; doi:10.3390/polym13101663)
Supplement: Supplementary file 1 [file polymers-13-01663-s001.zip › polymers-1225690-supplementary.pdf]

Supporting Information

Development of Bioinspired Functional Chitosan/Cellulose Nanofiber 3D Hydrogel Constructs by 3D Printing for Application in the Engineering of Mechanically Demanding Tissues

Arnaud Kamdem Tamo[1,2,3], Ingo Doench[1,2,3], Lukas Walter[1,2,3], Alexandra Montembault[4], Guillaume Sudre[4], Laurent David[4], Aliuska Morales-Helguera[5], Mischa Selig[6], Bernd Rolauffs[6], Anke Bernstein[6], Daniel Hoenders[7], Andreas Walther[7], Anayancy Osorio-Madrazo[1,2,3,*]

[1] Institute of Microsystems Engineering IMTEK, Laboratory for Sensors, University of Freiburg, 79110 Freiburg, Germany; arnaud.kamdem@imtek.uni-freiburg.de (A.K.T.); ingo.doench@imtek.uni-freiburg.de (I.D.); lukas.walter@imtek.uni-freiburg.de (L.W.)
[2] Freiburg Materials Research Center—FMF, University of Freiburg, 79104 Freiburg, Germany
[3] Freiburg Center for Interactive Materials and Bioinspired Technologies—FIT, University of Freiburg, 79110 Freiburg, Germany
[4] Ingénierie des Matériaux Polymères IMP UMR 5223—CNRS, Université Claude Bernard Lyon 1, Université de Lyon, 69622 Villeurbanne Cedex, France; alexandra.clayer-montembault@univ-lyon1.fr (A.M.); guillaume.sudre@univ-lyon1.fr (G.S.); laurent.david@univ-lyon1.fr (L.D.)
[5] Chemical Bioactive Center CBQ, Molecular Simulation and Drug Design Group, Central University of Las Villas, 50400 Santa Clara, Cuba; aliuska@uclv.edu.cu
[6] Center for Tissue Replacement, Regeneration & Neogenesis—G.E.R.N., Department of Orthopedics and Trauma Surgery, University of Freiburg, 79108 Freiburg, Germany; mischa.selig91@gmail.com (M.S.); berndrolauffs@googlemail.com (B.R.); anke.bernstein@uniklinik-freiburg.de (A.B.)
[7] Department of Chemistry, University Mainz, 55128 Mainz, Germany; daniel.hoenders@uni-mainz.de (D.H.); andreas.walther@uni-mainz.de (A.W.)
* Correspondence: anayancy.osorio@imtek.uni-freiburg.de; Tel.: +49-761-203-67363

Keywords: hydrogel 3D printing; polymer composites; chitosan; cellulose nanofibers; X-ray synchrotron scattering; micromechanics; tissue engineering

1. Orientation of Cellulose Nanofibers. Hermans' Orientation Factor and Affine Model

The affine model, for reorientation of rigid rod-like crystals,[1, 2] was used to obtain the orientation distribution function. Figure S1 shows the fit with Lorentz function of the curve of azimuthal intensity around the $(200)_I$ diffraction ring of Cellulose I allomorph crystals of the cellulose nanofibers (CNFs) vs. azimuthal angle ϕ, after evaluating the 2D WAXS patterns obtained for the CHI/CNF printed hydrogel filaments in situ at the synchrotron beamline, at different strain values during stretching (Figure 6b).

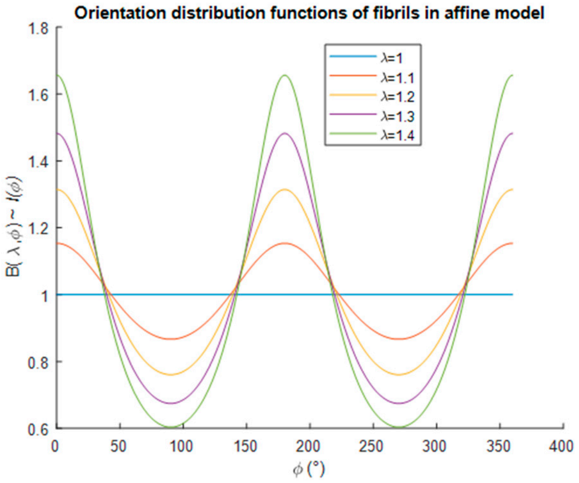

Figure S1. Azimuthal intensity around the diffraction signal $(200)_I$ of Cellulose I crystals constituting the cellulose nanofibers (CNFs) vs. azimuthal angle ϕ (Affine model, [1, 2]).

The Hermans' orientation factor is defined as follows (Equation (S1)):

$$f_H = \frac{3<cos^2\phi>-1}{2} \qquad \text{(S1)}$$

where <cos2ϕ> is the average cosine squared value for the (200)I diffraction ring. This latter is calculated using the Equation (S2):

$$< cos^2\phi \geq \frac{\sum_{i=0}^{90} I_i cos^2\phi_i sin\phi_i}{\sum_{i=0}^{90} I_i sin\phi_i} \qquad \text{(S2)}$$

In the manuscript Figure 6b (Right) the obtained Hermans orientation factor for the stretching of the hydrogel filament CHI3/CNF0.4 are shown, which fH values evolved from 0.002 to -0.110 when stretching till strain 24%, confirming the alignment of the CNFs to yield anisotropic hydrogel composites by stretching.

2. Optimization for High Size Resolution and Mechanical Performance of Printed CHI/CNF Hydrogels

Table S1. ANOVA analysis for printed hydrogel filament diameter.

Factor	ANOVA; R-sqr=.99998; Adj:.99992				
	**Sum of Square**	**Degree of Freedom**	**Mean Square**	**F-value**	***p***
(1) c(CHI)	8.33	1	8.33	20.39	0.046
(2) c(CNF)	1123.63	1	1123.63	2751.05	< 0.001
(3) Extrusion needle inner diameter (ID)	35892.72	1	35892.72	87878.41	< 0.001
1 by 3	174.01	1	174.01	426.04	0.002
2 by 3	37.80	1	37.80	92.56	0.012
Error	0.82	2	0.41		
Total SS	37237.31	7			

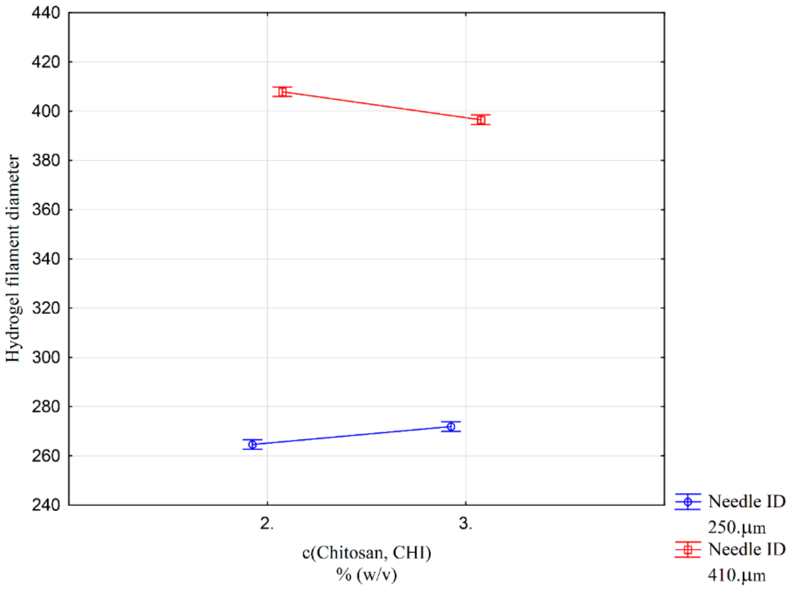

Figure S2. Effect of the interaction between c(CHI) and needle ID on the hydrogel filament diameter.

Table S2. ANOVA analysis for Young's modulus E of printed hydrogel filament.

Factor	ANOVA; R-sqr=.96862; Adj:.94508				
	**Sum of Square**	**Degree of Freedom**	**Mean Square**	**F-value**	***p***
(1) c(CHI)	3.1878125	1	3.1878125	85.7226891	< 0.001
(2) c(CNF)	0.8778125	1	0.8778125	23.605042	0.008
(3) Extrusion needle inner diameter (ID)	0.5253125	1	0.5253125	14.1260504	0.020
Error	0.14875	4	0.0371875		
Total SS	4.7396875	7			

Table S3. ANOVA analysis for stress at break of printed hydrogel filament.

Factor	ANOVA; R-sqr=.97112; Adj:.94946				
	**Sum of Square**	**Degree of Freedom**	**Mean Square**	**F-value**	***p***
(1) c(CHI)	0.500000	1	0.500000	73.26007	0.001
(2) c(CNF)	0.180000	1	0.180000	26.37363	0.007
(3) Extrusion needle inner diameter (ID)	0.238050	1	0.238050	34.87912	0.004
Error	0.027300	4	0.006825		
Total SS	0.945350	7			

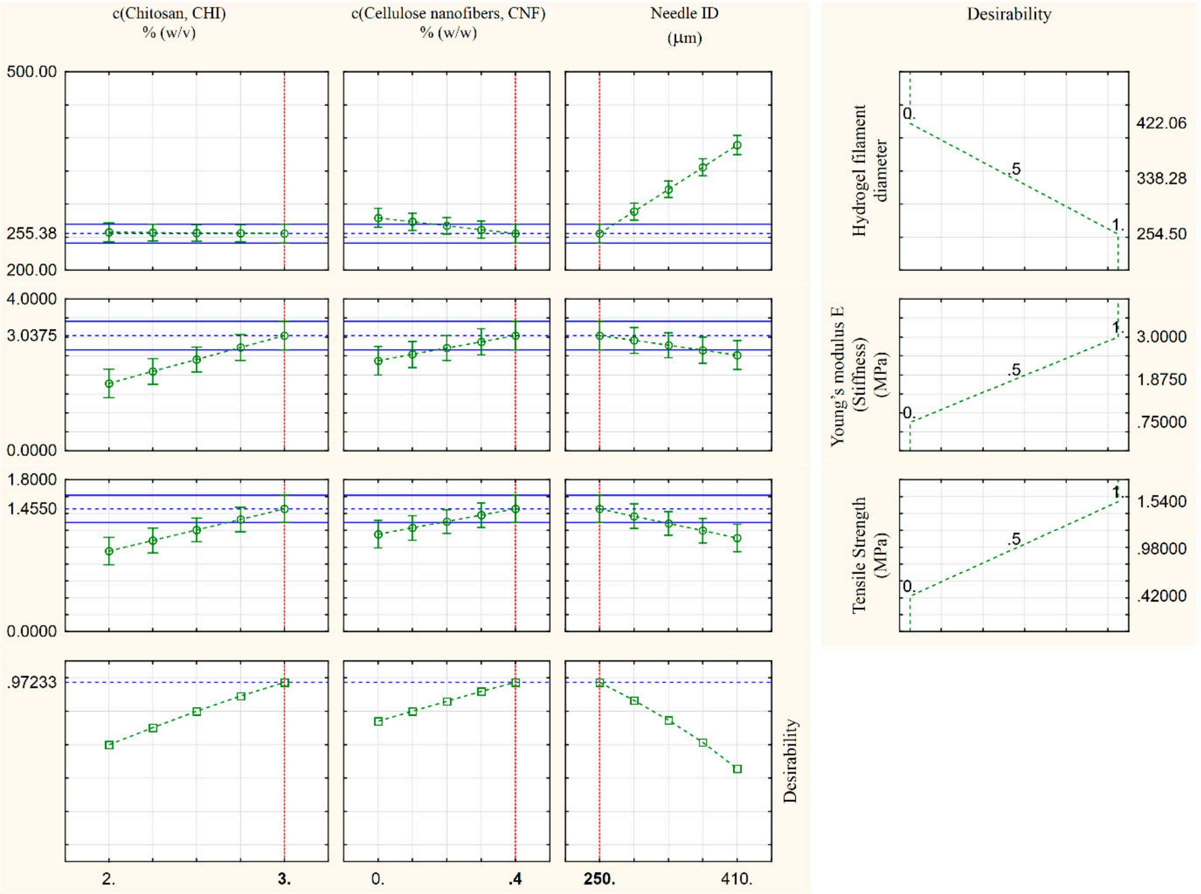

c(Chitosan, CHI) % (w/v)
c(Cellulose nanofibers, CNF) % (w/w)
Needle ID (µm)
Desirability
500.00
255.38
200.00
4.0000
3.0375
0.0000
1.8000
1.4550
0.0000
.97233
2.
3.
0.
.4
250.
410.
Desirability
Hydrogel filament diameter
422.06
338.28
254.50
Young's modulus E (Stiffness) (MPa)
3.0000
1.8750
.75000
Tensile Strength (MPa)
1.5400
.98000
.42000
0.
.5
1.

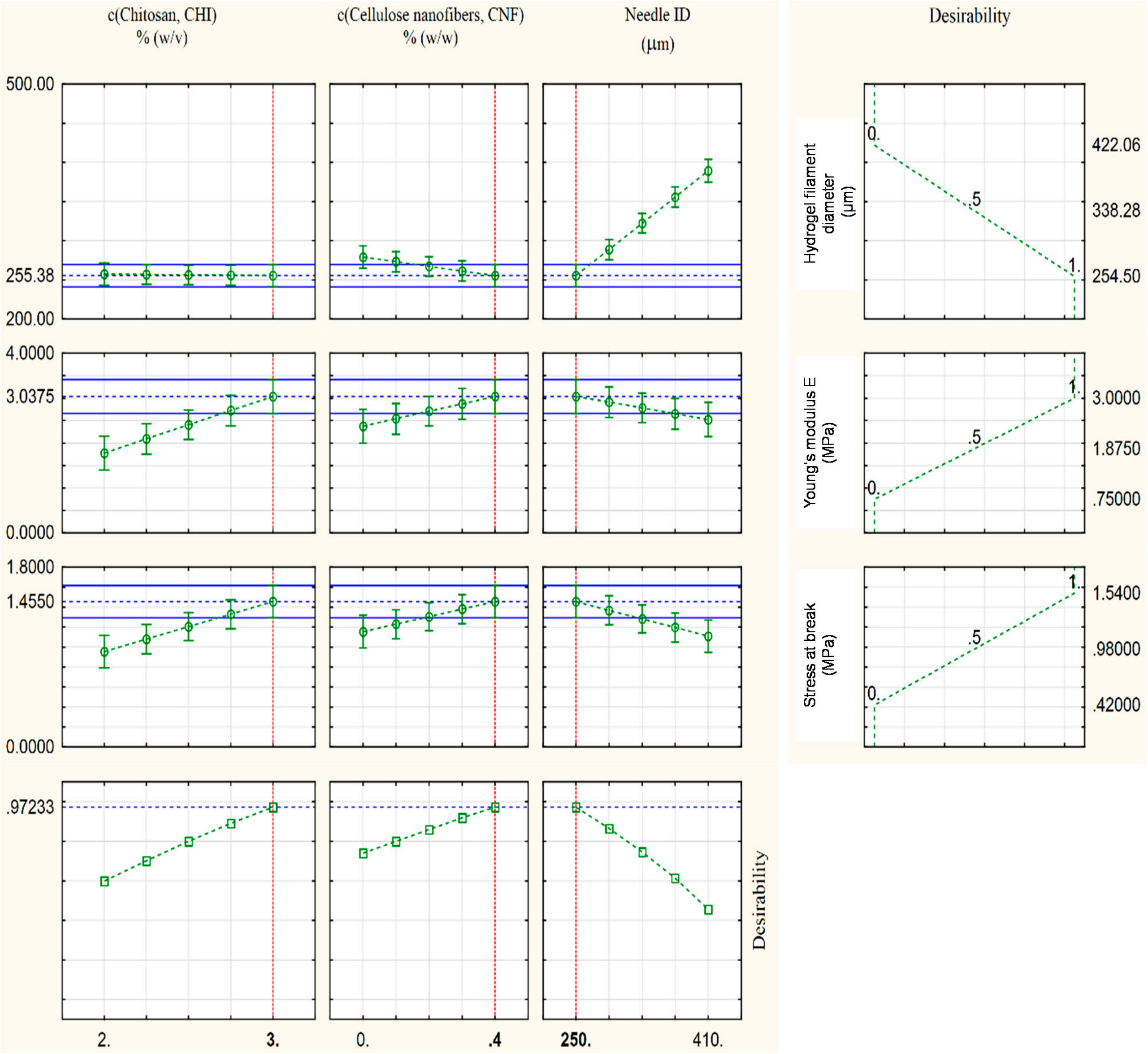

Figure S3. Profiles of predicted values and desirability.

3. Statistical analysis of LIVE and DEAD cells counting obtained from cell viability assays

Number of LIVE cells, and ratio of LIVE to DEAD cells (n(LIVE) : n(DEAD)) obtained in the culture of NIH/3T3 fibroblast in the hydrogel scaffolds (CHI2; CHI2/CNF0.4; CHI3) at Days 1, 3, and 6, were statistically analyzed. One-way Analysis of Variance (ANOVA) was performed using the software STATISTICA 10.0 (StatSoft Inc: Tulsa, USA, 2011), followed by the Tukey's HSD *post hoc* test if significant differences were found ($p < 0.05$) in ANOVA, as follows:

Analysis of Number of LIVE Cells :

1) For each formulation (CHI2, CHI2/CNF04 or CHI3), comparison at the different Days (1.00, 3.00, 6.00):

ANOVA

		Sum of Squares	df	Mean Square	F	Sig.
LIVE_CHI2	Between Groups	3255555.185	2	1627777.592	17.988	.001
	Within Groups	814434.421	9	90492.713		
	Total	4069989.606	11			
LIVE_CHI2/CNF04	Between Groups	4097595.547	2	2048797.773	13.493	.002

	Within Groups	1366546.008	9	151838.445		
	Total	5464141.555	11			
LIVE_CHI3	Between Groups	11924.622	2	5962.311	14.567	.002
	Within Groups	3683.602	9	409.289		
	Total	15608.225	11			

Multiple Comparisons

Tukey HSD

Dependent Variable	(I) Day	(J) Day	Mean Difference (I-J)	Std. Error	Sig.	95% Confidence Interval	
						Lower Bound	Upper Bound
LIVE_CHI2	1.00	3.00	-1262.15697*	212.71191	.001	-1856.0498	-668.2641
		6.00	-469.66895	212.71191	.123	-1063.5618	124.2239
	3.00	1.00	1262.15697*	212.71191	.001	668.2641	1856.0498
		6.00	792.48801*	212.71191	.012	198.5952	1386.3809
	6.00	1.00	469.66895	212.71191	.123	-124.2239	1063.5618
		3.00	-792.48801*	212.71191	.012	-1386.3809	-198.5952
LIVE_CHI2/CNF04	1.00	3.00	-1023.09180*	275.53443	.012	-1792.3855	-253.7981
		6.00	-1378.47191*	275.53443	.002	-2147.7656	-609.1782
	3.00	1.00	1023.09180*	275.53443	.012	253.7981	1792.3855
		6.00	-355.38010	275.53443	.435	-1124.6738	413.9136
	6.00	1.00	1378.47191*	275.53443	.002	609.1782	2147.7656
		3.00	355.38010	275.53443	.435	-413.9136	1124.6738
LIVE_CHI3	1.00	3.00	77.21431*	14.30540	.001	37.2735	117.1551
		6.00	38.16368	14.30540	.061	-1.7771	78.1044
	3.00	1.00	-77.21431*	14.30540	.001	-117.1551	-37.2735
		6.00	-39.05063	14.30540	.055	-78.9914	.8901
	6.00	1.00	-38.16368	14.30540	.061	-78.1044	1.7771
		3.00	39.05063	14.30540	.055	-.8901	78.9914

* The mean difference is significant at the 0.05 level.

2) For each Day (1.00, 3.00 or 6.00), comparison between the different formulations (CHI2, CHI2/CNF0.4 and CHI3):

ANOVA

		Sum of Squares	df	Mean Square	F	Sig.
LIVE_DIA1	Between Groups	18772.620	2	9386.310	3.388	.080
	Within Groups	24931.970	9	2770.219		
	Total	43704.590	11			
LIVE_DIA3	Between Groups	4477572.688	2	2238786.344	17.066	.001
	Within Groups	1180636.665	9	131181.852		
	Total	5658209.354	11			
LIVE_6	Between Groups	4080830.179	2	2040415.089	18.756	.001

	Within Groups	979095.397	9	108788.377		
	Total	5059925.575	11			

Multiple Comparisons

Tukey HSD

Dependent Variable	(I) Formulacion	(J) Formulacion	Mean Difference (I-J)	Std. Error	Sig.	95% Confidence Interval	
						Lower Bound	Upper Bound
LIVE_DIA1	CHI2(Y)	CHI2CNF04	81.05974	37.21706	.129	-22.8505	184.9700
		CHI3	86.48334	37.21706	.103	-17.4269	190.3936
	CHI2CNF04	CHI2(Y)	-81.05974	37.21706	.129	-184.9700	22.8505
		CHI3	5.42360	37.21706	.988	-98.4866	109.3338
	CHI3	CHI2(Y)	-86.48334	37.21706	.103	-190.3936	17.4269
		CHI2CNF04	-5.42360	37.21706	.988	-109.3338	98.4866
LIVE_DIA3	CHI2(Y)	CHI2CNF04	320.12490	256.10725	.456	-394.9280	1035.1778
		CHI3	1425.85461*	256.10725	.001	710.8017	2140.9075
	CHI2CNF04	CHI2(Y)	-320.12490	256.10725	.456	-1035.1778	394.9280
		CHI3	1105.72971*	256.10725	.005	390.6768	1820.7826
	CHI3	CHI2(Y)	-1425.85461*	256.10725	.001	-2140.9075	-710.8017
		CHI2CNF04	-1105.72971*	256.10725	.005	-1820.7826	-390.6768
LIVE_6	CHI2(Y)	CHI2CNF04	-827.74321*	233.22562	.015	-1478.9104	-176.5760
		CHI3	594.31597	233.22562	.073	-56.8513	1245.4832
	CHI2CNF04	CHI2(Y)	827.74321*	233.22562	.015	176.5760	1478.9104
		CHI3	1422.05919*	233.22562	.000	770.8920	2073.2264
	CHI3	CHI2(Y)	-594.31597	233.22562	.073	-1245.4832	56.8513
		CHI2CNF04	-1422.05919*	233.22562	.000	-2073.2264	-770.8920

* The mean difference is significant at the 0.05 level.

Analysis of LIVE to DEAD cells ratio n(LIVE):n(DEAD):

For each formulation (CHI2, CHI2/CNF04 or CHI3), comparison at the different Days (1.00, 3.00, 6.00):

ANOVA

		Sum of Squares	df	Mean Square	F	Sig.
CHI2	Between Groups	201.699	2	100.850	77.121	.000
	Within Groups	11.769	9	1.308		
	Total	213.468	11			
CHI2CNF04	Between Groups	4.315	2	2.158	4.090	.055
	Within Groups	4.748	9	.528		
	Total	9.063	11			
CHI3	Between Groups	6.108	2	3.054	58.993	.000
	Within Groups	.466	9	.052		

Total	6.574	11				

Multiple Comparisons

Tukey HSD

Dependent Variable	(I) Dia136	(J) Dia136	Mean Difference (I-J)	Std. Error	Sig.	95% Confidence Interval	
						Lower Bound	Upper Bound
CHI2	1.00	3.00	8.83086*	.80860	.000	6.5732	11.0885
		6.00	8.55658*	.80860	.000	6.2990	10.8142
	3.00	1.00	-8.83086*	.80860	.000	-11.0885	-6.5732
		6.00	-.27428	.80860	.939	-2.5319	1.9833
	6.00	1.00	-8.55658*	.80860	.000	-10.8142	-6.2990
		3.00	.27428	.80860	.939	-1.9833	2.5319
CHI2CNF04	1.00	3.00	1.34825	.51359	.065	-.0857	2.7822
		6.00	.16936	.51359	.942	-1.2646	1.6033
	3.00	1.00	-1.34825	.51359	.065	-2.7822	.0857
		6.00	-1.17889	.51359	.108	-2.6128	.2551
	6.00	1.00	-.16936	.51359	.942	-1.6033	1.2646
		3.00	1.17889	.51359	.108	-.2551	2.6128
CHI3	1.00	3.00	.99327*	.16088	.000	.5441	1.4425
		6.00	1.74182*	.16088	.000	1.2926	2.1910
	3.00	1.00	-.99327*	.16088	.000	-1.4425	-.5441
		6.00	.74856*	.16088	.003	.2994	1.1977
	6.00	1.00	-1.74182*	.16088	.000	-2.1910	-1.2926
		3.00	-.74856*	.16088	.003	-1.1977	-.2994

* The mean difference is significant at the 0.05 level.

For each Day (1.00, 3.00 or 6.00), comparison between the different formulations (CHI2, CHI2/CNF0.4 and CHI3):

ANOVA

		Sum of Squares	df	Mean Square	F	Sig.
Dia_1	Between Groups	139.231	2	69.616	46.714	.000
	Within Groups	13.412	9	1.490		
	Total	152.644	11			
Dia_3	Between Groups	3.433	2	1.717	14.838	.001
	Within Groups	1.041	9	.116		
	Total	4.474	11			
Dia_6	Between Groups	19.524	2	9.762	34.735	.000
	Within Groups	2.529	9	.281		
	Total	22.053	11			

Multiple Comparisons

Tukey HSD

Dependent Variable	(I) Formu	(J) Formu	Mean Difference (I-J)	Std. Error	Sig.	95% Confidence Interval	
						Lower Bound	Upper Bound
Dia_1	CHI2(Y)	CHI2CNF04	6.35827*	.86321	.000	3.9482	8.7684
		CHI3	7.85793*	.86321	.000	5.4478	10.2680
	CHI2CNF04	CHI2(Y)	-6.35827*	.86321	.000	-8.7684	-3.9482
		CHI3	1.49966	.86321	.244	-.9104	3.9098
	CHI3	CHI2(Y)	-7.85793*	.86321	.000	-10.2680	-5.4478
		CHI2CNF04	-1.49966	.86321	.244	-3.9098	.9104
Dia_3	CHI2(Y)	CHI2CNF04	-1.12434*	.24051	.003	-1.7958	-.4528
		CHI3	.02034	.24051	.996	-.6512	.6918
	CHI2CNF04	CHI2(Y)	1.12434*	.24051	.003	.4528	1.7958
		CHI3	1.14468*	.24051	.003	.4732	1.8162
	CHI3	CHI2(Y)	-.02034	.24051	.996	-.6918	.6512
		CHI2CNF04	-1.14468*	.24051	.003	-1.8162	-.4732
Dia_6	CHI2(Y)	CHI2CNF04	-2.02895*	.37486	.001	-3.0756	-.9823
		CHI3	1.04318	.37486	.051	-.0034	2.0898
	CHI2CNF04	CHI2(Y)	2.02895*	.37486	.001	.9823	3.0756
		CHI3	3.07213*	.37486	.000	2.0255	4.1187
	CHI3	CHI2(Y)	-1.04318	.37486	.051	-2.0898	.0034
		CHI2CNF04	-3.07213*	.37486	.000	-4.1187	-2.0255

* The mean difference is significant at the 0.05 level.

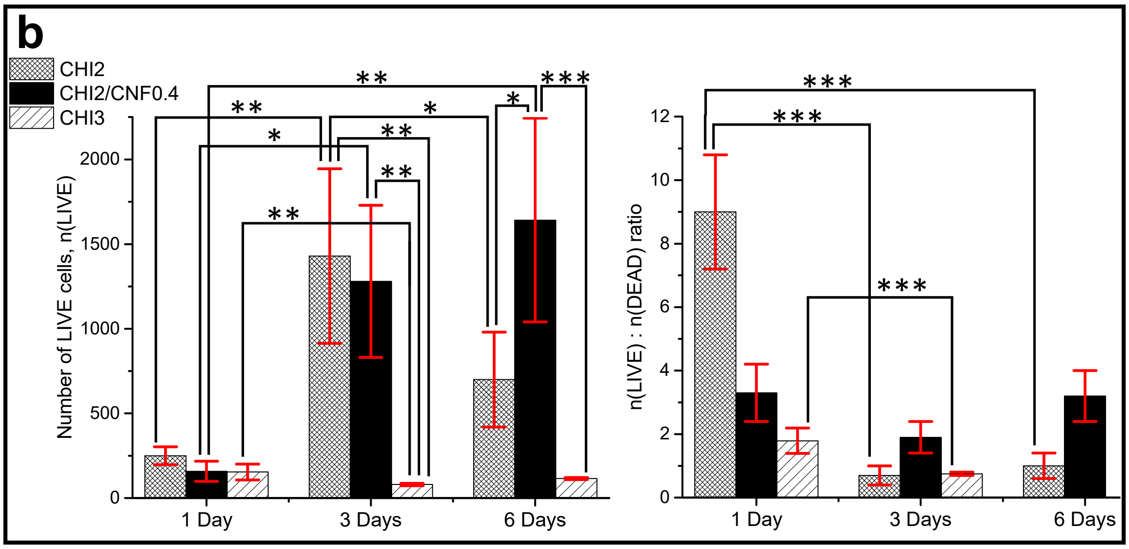

Figure 9b of Manuscript. (Left) Number of LIVE cells n(LIVE) (Left), (Right) n(LIVE)/n(DEAD) cell ratio obtained for the different CHI/CNF formulations at the different Days, expressed as means ± SDs, n =4 (*p < .05, **p < .01, ***p < .001).

Funding Sources

This research was supported by the Emmy Noether Programme of the German Research Foundation DFG (Grant number: OS 497/6-1).

References

1. Crawford, S.M.; Kolsky, H. Stress Birefringence in Polyethylene, Proceedings of the Physical Society. Section B 64(2) (1951) 119-125.
2. Boote, C.; Sturrock, E.J.; Attenburrow, G.E.; Meek, K.M. Psuedo-affine behaviour of collagen fibres during the uniaxial deformation of leather, *Journal of Materials Science* **2002**, *37*, 3651-3656.
